# Restoration of Spermatogenesis is Dependent on Activation of a SPRY4-ERK Checkpoint Following Germline Stem Cell Damage

**DOI:** 10.1101/2025.10.12.681919

**Published:** 2025-10-14

**Authors:** Ying Liu, Tansol Choi, Brad Pearson, Ryan Nachman, Whitney Woo, Na Xu, Ryan Schreiner, Romulo Hurtado, Marco Seandel, Shahin Rafii, Todd R. Evans

**Affiliations:** 1 Department of Surgery, Weill Cornell Medicine, New York, NY, USA; 2 Division of Regenerative Medicine, Hartman Institute for Therapeutic Organ Regeneration, Ansary Stem Cell Institute, Department of Medicine, Weill Cornell Medicine, New York, NY, USA; 3 Natural Sciences Department, LaGuardia Community College, Long Island City, New York, NY, USA

## Abstract

Mammalian spermatogonial stem cells (SSCs) sustain male fertility through continuous self-renewal and differentiation, leading to the production of haploid spermatozoa throughout adulthood. However, SSCs are vulnerable to genotoxic drugs, and patients receiving chemotherapy face a high risk of germline instability and infertility. The molecular mechanisms and cellular pathways that choreograph SSC recovery after chemotherapeutic insult remain unknown. Previously, we identified SPRY4 as an ERK-dependent negative feedback regulator of growth factor signaling that is critical for preservation of stem cell activity in cultured mouse SSCs. Here, we demonstrate that following alkylating agent busulfan (BU)-induced injury, germline-specific *Spry4* gene deletion (*Spry4*^G-KO^) reduces stem cell regeneration but promotes differentiation with rapidly enhanced nuclear ERK1/2 activity in undifferentiated (A_undiff_) spermatogonia (including SSCs) in adult mice. Genes essential for stem cell maintenance, including *Id1* and *Cxcl12*, were dysregulated by loss of *Spry4*. Furthermore, the MEK1/2 inhibitor PD0325901, but not mTORC1 inhibitor Rapamycin, was sufficient to promote spermatogonial proliferation in *Spry4*^G-KO^ testis 10 days post-BU treatment. Notably, the restoration of both spermatogonia pool and fertility was delayed in adult *Spry4*^G-KO^ males long-term after injury. In summary, germline-specific deletion of *Spry4* results in hyper-activation of the MAPK/ERK pathway in A_undiff_ spermatogonia, unleashing excessive spermatogenesis after germline damage, and ultimately impairing germline regeneration in adult males. Our study indicates an essential role for SPRY4-ERK signaling as a molecular checkpoint in securing SSC recovery upon chemotherapy drug-induced germline damage, revealing how stem cells normally withstand environmental stress.

## Introduction

In adult mammalian testis, haploid spermatozoa are produced from unipotent spermatogonial stem cells (SSCs) through a tightly regulated process known as spermatogenesis. SSCs, which reside as type A-single spermatogonia (A_s_) in the Sertoli cell-based niche at the basal lamina of the seminiferous tubules, are capable of both self-renewal and differentiation to maintain the germline stem cell pool while generating differentiated progeny throughout adulthood. Upon fate commitment, A_s_ spermatogonia undergo a series of incomplete mitotic divisions to form A_paired_ (A_pr_) spermatogonia consisting of two interconnected cells, and then A_aligned_ (A_al_) spermatogonia consisting of 4, 8, or 16 interconnected cells in chains. A_s_, A_pr_, and A_al_ spermatogonia together constitute the undifferentiated spermatogonia (A_undiff_) pool, with A_al_ spermatogonia ultimately transitioning into differentiated spermatogonia (A_diff_) and proceeding to meiosis^1,2^.

Glial cell line-derived neurotrophic factor (GDNF) and fibroblast growth factor (FGF) are essential for SSC self-renewal and maintenance^3^. Both bind to receptor tyrosine kinases (RTKs) on the SSC membrane to activate the downstream effector Ras and initiate intracellular signaling cascades including the PI3K/AKT and MAPK/ERK pathways^4^. Both GDNF and FGF signaling regulate mTORC1, the central signaling hub that governs SSC fate commitment^3,5^. In adult mouse testis, mTORC1 activity is suppressed in most self-renewing spermatogonia but induced by the PI3K/AKT pathway to generate cell-cycle-activated progenitors^6–8^. The PI3K/AKT/mTOR signaling pathway has been extensively explored as the master regulator that drives SSC proliferation and differentiation in both cultured SSCs and mouse models^9–11^, although an *in vitro* study suggests that the MAPK/ERK pathway is also required to efficiently activate mTORC1^12^. The MAPK/ERK pathway can be rapidly activated in response to various extracellular or intracellular stimuli to regulate distinct cellular processes including proliferation, differentiation, and regeneration^13^. Following sequential phosphorylation along the Ras/Raf/MEK/ERK signaling cascade, phosphorylated ERK1/2 (p-ERK1/2) impacts a broad set of substrates localized within the cytoplasm, including mTORC1^14^. P-ERK1/2 can also rapidly translocate from the cytoplasm to the nucleus to regulate transcriptional activity. The subcellular localization bestows p-ERK1/2 with distinct functions in regulating cellular responses to stimuli^15,16^. Unlike PI3K/AKT and mTORC1, the function of the MAPK/ERK pathway in SSCs remains elusive. Several studies reported that both GDNF and FGF2 can activate the MAPK/ERK pathway and promote SSCs proliferation *in vitro*^10,17,18^, although FGF2 has also been suggested to promote cultured SSCs to a more differentiation-primed state^19^. Studies of A_undiff_ spermatogonia within adult mouse testis indicate that intrinsic MAPK/ERK pathway components and regulators play a role in A_undiff_ spermatogonia self-renewal^20,21^, although MAPK/ERK signaling from the somatic niche has also been shown to contribute to the cyclical activity of SSCs^21^.

Like many types of adult stem cells, SSCs are very sensitive to chemotherapy drugs including the alkylating agent busulfan (BU), which is particularly toxic to SSCs and other rapidly dividing spermatogonia^22,23^. However, a few A_undiff_ spermatogonia can escape from low-dose BU treatment, repopulate rapidly after genotoxic stress, regenerate the seminiferous epithelium, and restore long-term fertility^24^. Although the physiological and molecular features of regenerative spermatogonia have been characterized in adult mice^25–27^, how the germline-intrinsic signaling modulators respond to injury and cooperate to restore germline homeostasis remains obscure.

We identified SPRY4 (Sprouty4), primarily known as a negative feedback regulator of RTK-MAPK/ERK pathway through its interference with Ras activation^28,29^, as a hallmark protein that is exclusively expressed in A_undiff_ spermatogonia within adult mouse germ cells^30^. In cultured SSCs, SPRY4 modulates the cellular response to FGF2 or GDNF through the MAPK/ERK pathway, with *Spry4* deletion enhancing early differentiation and diminishing stem cell activity in transplantation analysis. However, germline-specific *Spry4* deletion from adult mice (*Spry4*^G-KO^) does not affect stem cell colonization and differentiation when tested by transplanting total testicular cells to wild-type recipients^30^. To further address the SPRY4 function in the regeneration of SSCs *in vivo*, we employed a BU-induced germline damage and recovery mouse model to uncover the signaling pathways choreographing stem cell homeostasis after injury.

Toward this end, adult *Spry4*^G-KO^ or wild-type (*Spry4*^WT^) males were treated with a single low dose of BU, and SSC recovery and differentiation were temporally and spatially determined. We found that quiescent SSCs within *Spry4*^WT^ mouse testis recovered from injury through a series of well-orchestrated transitions to achieve cell cycle activation and fate commitment. Notably, we found that germline-intrinsic SPRY4-ERK signaling must be carefully titrated to appropriately initiate stem cell regeneration. However, loss of *Spry4* resulted in hyperactivation of the MAPK/ERK pathway in A_undiff_ spermatogonia, which induced transient stem cell proliferation rapidly after damage and promoted untimely differentiation. The transition from proliferating to fate-primed A_undiff_ spermatogonia was correlated with p-ERK1/2 translocation from the cytoplasm to the nucleus. We also show that young adult *Spry4*^G-KO^ males experience a fertility defect with delayed recovery of A_undiff_ spermatogonia pool after BU treatment, suggesting that dysregulation of the MAPK/ERK pathway is detrimental to SSC homeostasis during regeneration. Therefore, germline-intrinsic SPRY4-ERK signaling is essential to maintain and prevent exhaustion of SSCs after injury, thereby ensuring long-term spermatogenesis under environmental stresses through adulthood.

## Results

### Adult males with short-term germline-specific *Spry4* deletion can undergo spermatogenesis at steady state

We have previously demonstrated that mouse SSCs respond robustly to GDNF or FGF2 induction in culture and activate *Spry4* expression to modulate the MAPK/ERK pathway^30^. Loss of *Spry4* promoted *in vitro* differentiation of SSCs at the expense of self-renewal. To investigate the *in vivo* cell autonomous function of SPRY4 under normal conditions, specifically in adult mice, we crossed *G**frα1*-*C**reER*^*T2*^*;**R**osa26-LSL-td**T**omato* (referred to as GCRT) mice with *Spry4*^*flox/flox*^ mice to create GCRT;*Spry4*^*flox/flox*^ mice^30^. Germline-specific *Spry4* deletion (referred to as *Spry4*^G-KO^) was generated by injecting tamoxifen (100 mg/kg) intraperitoneally for two weeks in adult (2- to 4-month-old) GCRT;*Spry4*^*flox/flox*^ mice. Tamoxifen effectively activates *Cre* in spermatogonia, which then pass the genetic modification to all stages of differentiating germline descendants in the testis^31^. As expected, robust expression of tdTomato, a stop-floxed lineage tracer, was observed only within the *Spry4*^G-KO^ mouse testis (Figure S1A). Whole-mount immunofluorescence (IF) staining revealed that these tdTomato^+^ cells expressed many germ cell markers, including VASA (marking all germ cells), GFRα1(delineating the most primitive set of A_undiff_ spermatogonia), and MCAM/CD146 (delineating both A_undiff_ spermatogonia and progenitors^32,33^), but not Vimentin (marking Sertoli cells, the somatic niche of spermatogonia). These data illustrate efficient gene deletion of *Spry4* within different stages of germ cells (Figure S1B – 1D).

We then collected testes for analysis from *Spry4*^G-KO^ or control *Spry4*^*flox/flox*^ (referred to as *Spry4*^WT^) mice 6 weeks after the first tamoxifen injection (harvested within 4 months of age) for analysis. No marked difference in testis size (Figure S2A) or mature spermatozoa count (Figure S2B) was identified between *Spry4*^G-KO^ and *Spry4*^WT^ testes. Histological analysis revealed that seminiferous tubules in the testis sections from both *Spry4*^G-KO^ and *Spry4*^WT^ mice were populated with germ cells at different developmental stages, and functionally mature spermatozoa were identified in the cauda epididymis of both genotypes (Figure S2C). Taken together, GFRα1-CreER^T2^ induced germline-specific *Spry4* deletion does not significantly affect spermatogenesis in adult mouse testis in the short-term (6 weeks post-tamoxifen), suggesting that SPRY4 is dispensable for spermatogonia differentiation at steady state in young adulthood.

### *Spry4* deletion reduces stem cell regeneration in chemotherapy damaged adult mouse testis

**Figure 1.**
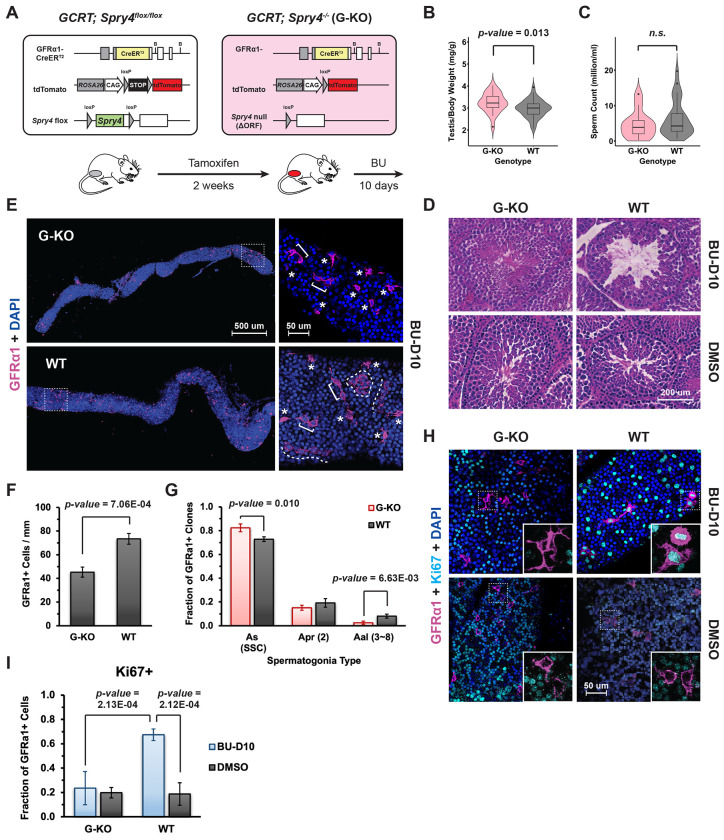
Following BU-induced damage, germline-specific *Spry4* deletion reduces proliferation of A_undiff_ spermatogonia in adult mouse testis. **(A)** Schema illustrating the induction of germline-specific *Spry4* deletion and germline recovery in response to low dose BU treatment in adult mouse testis. GCRT, *G**fra1*-*C**reER*^*T2*^*;**R**osa26-LSL-td**T**omato*. Testes collected from adult G-KO or WT mice 10 days post-BU treatment were compared for **(B)** testis to body weight ratio and **(C)** sperm count. Data are mean (SD), n>10 mice analyzed per genotype. **(D)** Hematoxylin and eosin (H&E)–stained histological cross-sections of testes collected from adult *Spry4*^G-KO^ (G-KO) or *Spry4*^WT^ (WT) mice at 10 days post BU (BU-D10, Top) or post DMSO (DMSO, Bottom) treatment. Lower magnification images are shown in Fig S3. **(E)** Representative whole-mount immunofluorescence (IF) images illustrating GFRα1^+^ populations in tubules from *Spry4*^G-KO^ and *Spry4*^WT^ mice at BU-D10. Higher magnification images of indicated regions are shown on the right. Magenta, GFRα1; Blue, DAPI. Asterisks, A_s_ spermatogonia; Brackets, A_pr_ spermatogonia; Dashed lines, A_al_ spermatogonia (4 – 8 cells). **(F)** Quantification of GFRα1^+^ cells per mm of seminiferous tubules. **(G)** Fraction of A_s_, A_pr_, and A_al_ spermatogonia based on whole-mount IF analysis of GFRα1^+^ clones. Number in round brackets indicate cell numbers per GFRα1^+^ clone. Data are mean (SD), n>3 mice analyzed per genotype. **(H)** Whole-mount IF staining on testicular tubules collected after BU-D10 or DMSO treatment. Higher magnification images of indicated regions are shown in the square. Magenta, GFRα1; Cyan, Ki67; Blue, DAPI. Inset, higher magnification images of indicated regions without DAPI. **(I)** Quantification of Ki67^+^ cells within GFRα1^+^ cells in whole-mount IF (n>4 mice analyzed per condition, n>100 GFRα1^+^ cells scored per animal).

To investigate Spry4 function in accelerated stem cell regeneration *in vivo*, we used a chemotherapy-induced germline damage and recovery model to explore whether loss of *Spry4* impacts stem cell activity during germline recovery^27^. The alkylating agent busulfan (BU) has been reported to cause apoptosis of undifferentiated (A_undiff_) and differentiating (A_diff_) spermatogonia in rodents^34^. Although the highest non-lethal BU dose (40 mg/kg) results in infertility with few spermatogonia remaining in the seminiferous tubules, lower doses of BU (e.g., 10 mg/kg) preserve some A_undiff_ spermatogonia that can restore the germline, providing a reliable model of spermatogenesis that requires SSC regeneration and differentiation^27,34^. Therefore, we used a single low dose of BU (10 mg/kg) to induce germline damage in adult *Spry4*^G-KO^ or *Spry4*^WT^ males and analyzed testes after recovering from BU treatment for 10 days (BU-D10), a latency period sufficient for surviving A_undiff_ spermatogonia to actively proliferate and initiate regeneration (Figure 1A).

We found that testes collected from *Spry4*^G-KO^ males were significantly larger than those from *Spry4*^WT^ males at BU-D10 (Figure 1B), although both *Spry4*^G-KO^ and *Spry4*^WT^ mice generated similar sperm counts (Figure 1C). Histological analysis of the testis sections collected at BU-D10 showed that the seminiferous tubules within *Spry4*^G-KO^ mouse testes contained germ cells at different developmental stages, similar to the tubules of *Spry4*^G-KO^ or *Spry4*^WT^ mice with control DMSO treatment (DMSO) (Figure 1D and S3). In contrast, most seminiferous tubules in the testis sections of *Spry4*^WT^ mice were degenerated with considerable loss of developing germ cells and mature spermatozoa, a phenotype which has been reported in low dose BU treated males^34^ (Figure 1D and S3).

By whole-mount IF staining of seminiferous tubules, we identified GFRα1^+^ cells in testes collected from both *Spry4*^G-KO^ and *Spry4*^WT^ mice at BU-D10 (Figure 1E). Comparing to *Spry4*^WT^ mice post-BU, the tubules collected from *Spry4*^G-KO^ mice contained less GFRα1^+^ cells (Figure 1F), with over 82% of GFRα1^+^ cells existing as A_s_ spermatogonia, the most primitive spermatogonia resembling SSCs (Figure 1E, asterisks). The fractions of A_s_ spermatogonia within the *Spry4*^G-KO^ tubules were significantly higher than those identified in the tubules collected from *Spry4*^WT^ mice (Figure 1G). A_s_ spermatogonia can divide to form two spermatogonia connected by an intercellular bridge (A_pr_), then proceed to form a chain of aligned (A_al_) spermatogonia which are considered progenitors lacking regenerative capacity. We identified over 15% of A_pr_ spermatogonia clones and numerous chains of A_al_ spermatogonia containing up to 8 GFRα1^+^ cells within *Spry4*^WT^ tubules (Figure 1E and 1G, WT). However, A_al_ spermatogonia chains with 8 GFRα1^+^ cells were barely detected within the tubules collected from *Spry4*^G-KO^ mice (Figure 1E and 1G, G-KO).

To evaluate regenerative capacity in A_undiff_ spermatogonia, we co-stained cell proliferation marker Ki67 with GFRα1. We found that only about 20% of GFRα1^+^ cells were positive for Ki67 in the tubules collected from *Spry4*^G-KO^ or *Spry4*^WT^ mice without chemotherapeutic insult (DMSO), suggesting that most A_undiff_ spermatogonia remain quiescent without damage (Figure 1H and 1I, DMSO). However, nearly 70% of *Spry4*^WT^ GFRα1^+^ cells surviving BU treatment were highly proliferative with strong Ki67 staining at BU-D10 (Figure 1H and 1I, WT, BU-D10), as has been reported in regenerating spermatogonia^27^. Notably, only about 25% of *Spry4*^G-KO^ GFRα1^+^ cells were actively proliferating at BU-D10, suggesting that spermatogonia regeneration is repressed in *Spry4*^G-KO^ mice after chemotherapy drug-induced germline damage (Figure 1H and 1I, G-KO, BU-D10).

### *Spry4*^G-KO^ spermatogonia exhibit enhanced differentiation during germline recovery

**Figure 2.**
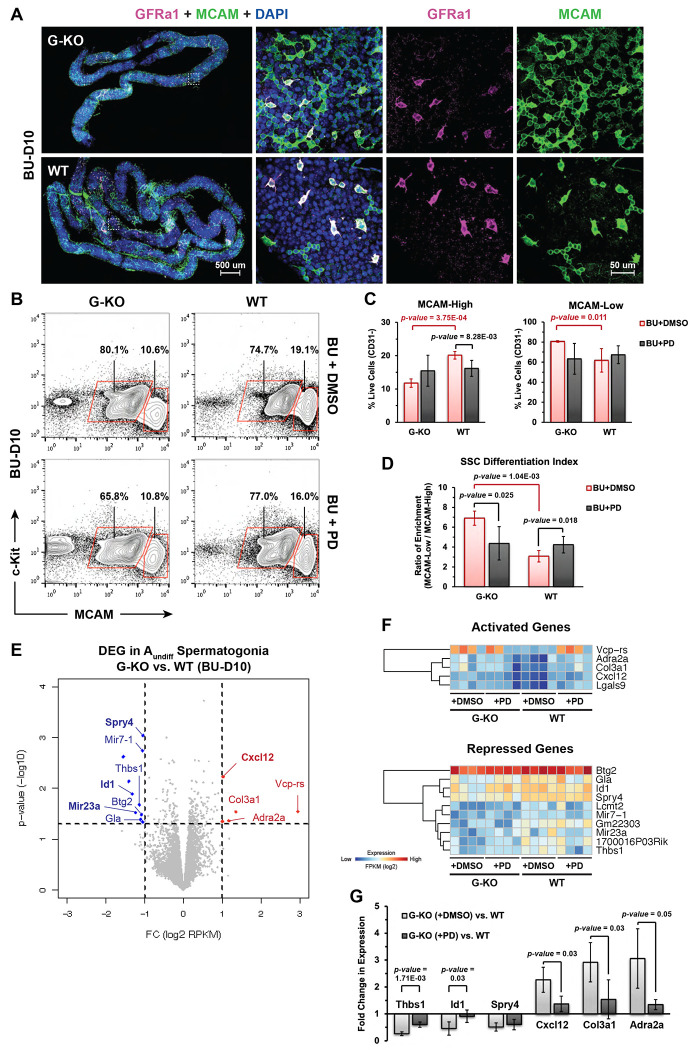
*Spry4*^G-KO^ spermatogonia exhibit enhanced differentiation with transcriptional changes at selected genes during germline regeneration (BU-D10). **(A)** Representative wholemount IF images illustrating GFRα1^+^ and MCAM^+^ populations in tubules from *Spry4*^G-KO^ and *Spry4*^WT^ mice at BU-D10. Higher magnification images of indicated regions are shown on the right. Magenta, GFRα1; Green, MCAM; Blue, DAPI. **(B)** Fluorescence-activated cell sorting (FACS) strategy for isolation of MCAM^High^c-Kit^−^ (A_undiff_) and MCAM^Low^ (A_diff_) spermatogonia. **(C)** Percentage of cells within gates are indicated (n>3 mice analyzed per condition). **(D)** SSC Differentiation Index (SDI) calculated by the ratio of enrichment between MCAM^Low^ (A_diff_) and MCAM^High^c-Kit^−^ (A_undiff_) spermatogonia seen in Fig. 2C. **(E)** Volcano plot of expression profiles comparing MCAM^High^c-Kit^−^ population isolated from *Spry4*^G-KO^ or *Spry4*^WT^ mouse testes at BU-D10. Selected genes were highlighted based on expression changes: Red, genes upregulated in *Spry4*^G-KO^ mice; Blue, genes downregulated in *Spry4*^G-KO^ mice. (n=4 mice analyzed per condition) **(F)** Expression of genes activated or suppressed in *Spry4*^G-KO^ mice. Red and blue indicate relatively high and low gene expression, respectively. **(G)** qRT-PCR analysis of selected genes as highlighted in Fig. 2E. Data are mean (SD), n=4 mice analyzed per condition.

To characterize spermatogonia populations within the seminiferous tubules of BU-damaged testis, we applied whole-mount IF staining for MCAM to detect both A_undiff_ and A_diff_ spermatogonia within the tubules collected from *Spry4*^G-KO^ or *Spry4*^WT^ mouse testes at BU-D10. Although GFRα1^+^ cells (A_undiff_ spermatogonia) were found more in the tubules of *Spry4*^WT^ mice than in *Spry4*^G-KO^ mice (Figure 1F), MCAM^+^ cells were sparse in multiple tubules collected from *Spry4*^WT^ mice, with some regions completely devoid of GFRα1^−^MCAM^+^ cells (A_diff_ spermatogonia), corresponding with the initiation of regeneration post damage (Figure 2A, WT). Strikingly, the tubules collected from *Spry4*^G-KO^ mice were nearly entirely populated with GFRα1^−^MCAM^+^ cells, revealing spermatogonial hyper-differentiation during germline recovery (Figure 2A, G-KO).

We further quantified the spermatogonia by fluorescence-activated cell sorting (FACS) of MCAM^+^ cells, with the high MCAM and negative c-Kit (MCAM^High^c-Kit^−^) population enriched for A_undiff_ spermatogonia and low MCAM (MCAM^Low^) population enriched for A_diff_ spermatogonia (Figure S4A). We observed tdTomato signal in most MCAM^High^c-Kit^−^ cells isolated from *Spry4*^G-KO^ mouse testes, confirming efficient GFRα1-Cre^ERT2^-induced recombination within A_undiff_ spermatogonia (Figure S4B). Notably, the MCAM^High^c-Kit^−^ population isolated from *Spry4*^G-KO^ mice was significantly reduced compared to those isolated from *Spry4*^WT^ mice at BU-D10 (Figure 2B and 2C, MCAM-High, BU+DMSO). Compared with *Spry4*^WT^ mice, the MCAM^Low^ population containing more differentiating spermatogonia was significantly increased in *Spry4*^G-KO^ mouse testes (Figure 2B and 2C, MCAM-Low, BU+DMSO). To quantify differentiation status in testicular cells, we defined the SSC Differentiation Index (SDI) by calculating the ratio of enrichment between MCAM^Low^ (A_diff_) and MCAM^High^c-Kit^−^ (A_undiff_) spermatogonia (See Methods). The SDI indicated that the *Spry4*^G-KO^ spermatogonia retain a more differentiated status compared to the *Spry4*^WT^ spermatogonia at BU-D10 (Figure 2D, BU+DMSO).

SPRY4 has been identified as a negative feedback regulator of the MAPK/ERK pathway. Our prior studies with cultured adult mouse SSCs revealed that SPRY4 is critical for preservation of stem cell activity through ERK-dependent regulation of FGF and GDNF signaling. To investigate the function of SPRY4-ERK signaling during chemotherapy-induced germline damage and recovery *in vivo*, ERK activity in *Spry4*^G-KO^ or *Spry4*^WT^ mice was blocked by treating mice daily with the MEK inhibitor PD0325901 (PD, also known as mirdametinib) prior to regeneration (3 days after low dose BU treatment) and analyzed at BU-D10. Notably, PD treatment resulted in similar MCAM^High^c-Kit^−^ or MCAM^Low^ populations in *Spry4*^G-KO^ and *Spry4*^WT^ mice, while the MCAM^High^c-Kit^−^ population collected from *Spry4*^WT^ mice was slightly reduced with PD treatment (Figure 2B and 2C, BU+PD). Corresponding with these findings, the SDI of PD-treated *Spry4*^G-KO^ spermatogonia is substantially reduced to the levels seen in *Spry4*^WT^ spermatogonia, suggesting that PD treatment can restore the *Spry4*^G-KO^ spermatogonia to a less differentiated status (Figure 2D, BU+PD).

### Characterization of SPRY4-dependent genes in regenerating spermatogonia

To investigate the molecular mechanism underlying spermatogonia hyper-differentiation in *Spry4*^G-KO^ mice post-BU, we applied bulk RNA-seq to characterize *Spry4*-dependent genes in MCAM^High^c-Kit^−^ (A_undiff_) spermatogonia at BU-D10. Surprisingly, very few genes demonstrated significant differential expression patterns (p-value < 0.05 and |fold change of log2 FPKM| > 1) in the A_undiff_ spermatogonia FACS-isolated from *Spry4*^G-KO^ mouse testes compared to *Spry4*^WT^ (Figure 2E). As expected, transcripts for the *Spry4* gene were significantly reduced in the A_undiff_ spermatogonia collected from *Spry4*^G-KO^ mouse testes (Figure 2E – 2G). Although PD treatment down-regulated *Spry4* gene expression in the A_undiff_ spermatogonia collected from *Spry4*^WT^ mouse testes (Figure S5), reflecting the role of SPRY4 as a MAPK/ERK-dependent negative feedback regulator, no expression difference was identified from *Spry4*^G-KO^ spermatogonia with or without PD treatment (Figure 2F, 2G, and S5). Among the genes downregulated after *Spry4* deletion, *Id1* is critical for mammalian stem cell maintenance by promoting quiescence and preventing premature differentiation in multiple adult stem cells^35^. Notably, treating *Spry4*^G-KO^ mice with the MAPK/ERK pathway inhibitor PD restored *Id1* gene expression levels to those seen in *Spry4*^WT^ mice (Figure 2F, 2G, and S5). Besides the *Id1* gene, two microRNA genes, Mir7-1 and Mir23a, were downregulated in *Spry4*^G-KO^ spermatogonia (Figure 2E, 2F). Mir7-1 is one of three genomic loci encoding miR-7, a highly conserved miRNA playing key roles in both normal organ development and tumorigenesis^36^. Several studies in male subfertile patients indicate that the levels of mature guide strand of Mir23a may associate with spermatogenesis-related gene expression^37,38^. However, the functions of both microRNAs have not been investigated in SSCs. We also found that the expression of *Cxcl12*, a major regulator of spermatogonia survival and migration, was significantly upregulated in *Spry4*^G-KO^ A_undiff_ spermatogonia, with this effect largely diminished after PD treatment (Figure 2E – 2G, and S5).

### SPRY4 is required for A_undiff_ spermatogonia regeneration through inhibition of nuclear ERK activity

**Figure 3.**
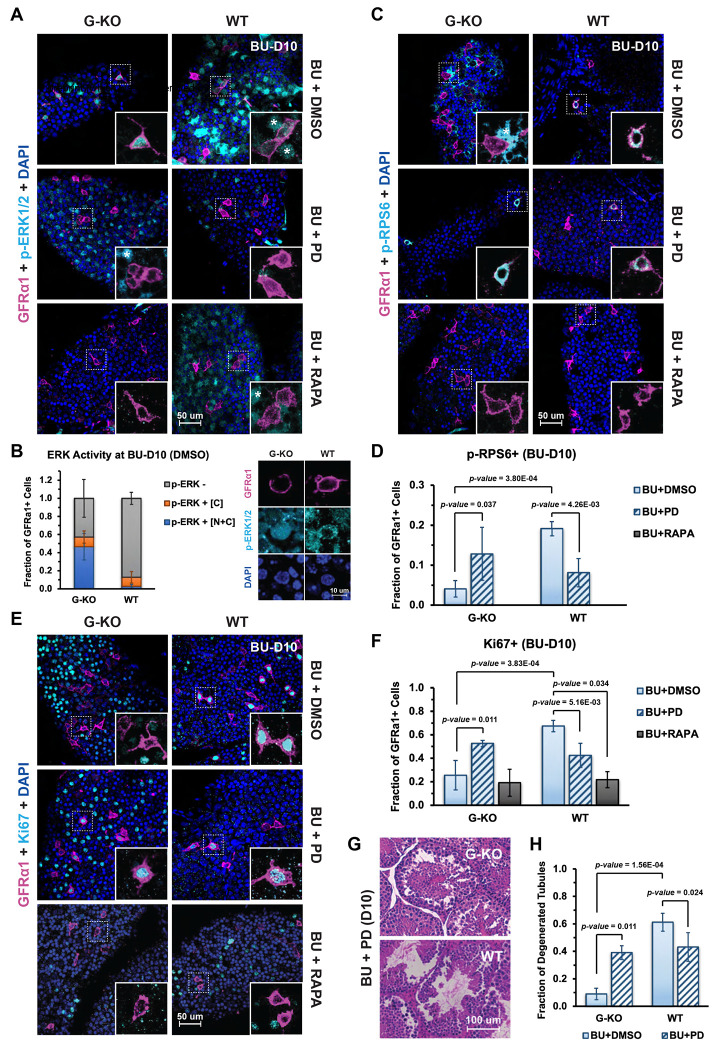
SPRY4 is required for A_undiff_ spermatogonia regeneration through inhibiting ERK signaling activity. Adult *Spry4*^G-KO^ or *Spry4*^WT^ mice were treated with single low dose BU and then daily with DMSO (BU+DMSO), PD0325901 (BU+PD), or Rapamycin (BU+RAPA) starting from day 3 post-BU. Testes were collected for analysis at 10 days post-BU by whole-mount IF staining for **(A)** ERK activity. Magenta, GFRα1; Cyan, p-ERK1/2; Blue, DAPI, or **(C)** mTORC1 activity. Magenta, GFRα1; Cyan, p-RPS6; Blue, DAPI, or **(E)** Proliferation. Magenta, GFRα1; Cyan, Ki67; Blue, DAPI. Inset, higher magnification images of indicated regions without DAPI. Asterisks, somatic cells. GFRα1^+^ cells in whole-mount IF were counted for the fraction of **(B)** p-ERK1/2 enrichment within both nucleus and cytoplasm (p-ERK^+^ [N+C]), only in the cytoplasm (p-ERK^+^ [C]), or no detectable p-ERK1/2 (p-ERK^−^) at BU-D10 (BU+DMSO), with higher magnification images of GFRα1^+^ cells in *Spry4*^G-KO^ or *Spry4*^WT^ tubules shown on the left, **(D)** p-RPS6^+^ cells, or **(F)** Ki67^+^ cells. n>3 mice analyzed per condition, n>100 GFRα1^+^ cells scored per animal. **(G)** H&E–stained histological cross-sections of adult mouse testes collected at 10 days post BU with PD0325901 treatment (BU+PD, D10). **(H)** Fraction of degenerated tubules identified in Fig. 3G. n=3 mice analyzed per condition. Data are mean (SD).

ERK1/2 can be activated by growth factors to p-ERK1/2 and translocate from cytoplasm to nucleus to modulate gene expression^16^. Our study with cultured SSCs revealed that SPRY4 is a critical regulator of MAPK/ERK signaling patterns during stem cell differentiation^30^. By whole-mount IF staining of *Spry4*^WT^ testicular tubules, we found that p-ERK1/2 was excluded from most spermatogonia, including GFRα1^+^ (A_undiff_) and GFRα1^−^MCAM^+^ (A_diff_) spermatogonia during regeneration (BU-D10) (Figure 3A and S6A, WT, BU+DMSO), with only about 10% of GFRα1^+^ spermatogonia slightly enriched for p-ERK1/2 in the cytoplasm or within the whole cells (Figure 3B, WT). By contrast, p-ERK1/2 was enriched in nearly 60% of GFRα1^+^ spermatogonia identified from *Spry4*^G-KO^ testicular tubules, and 47% of GFRα1^+^ spermatogonia contained p-ERK1/2 activity within the entire cell including the nuclei (Figure 3A, 3B and S6B, G-KO, BU+DMSO). Importantly, inhibiting the MAPK/ERK pathway with PD treatment was sufficient to eliminate p-ERK1/2 from the spermatogonia in both *Spry4*^G-KO^ and *Spry4*^WT^ testes, although p-ERK1/2 was still detected in many tdTomato^−^ or MCAM^−^ cells (Sertoli cells) within the seminiferous tubules (Figure 3A and S6, BU+PD, asterisks). Several studies have suggested that the MAPK/ERK pathway may regulate spermatogonia homeostasis through mTORC1, which has been reported as a critical inducer of A_undiff_ spermatogonia regeneration. However, we did not observe significant p-ERK1/2 in the spermatogonia of either *Spry4*^G-KO^ or *Spry4*^WT^ mice treated with Rapamycin (RAPA), the inhibitor of mTORC1 (Figure 3A and S6, BU+RAPA).

To detect mTORC1 activity within spermatogonia during regeneration, we analyzed phosphorylated ribosomal protein S6 (p-RPS6) by whole-mount IF staining of the seminiferous tubules collected at BU-D10. Consistent with previous studies^27^, we identified nearly 20% of GFRα1^+^ cells with strong cytoplasmic p-RPS6 in the tubules collected from *Spry4*^WT^ mice at BU-D10 (Figure 3C and 3D, WT, BU+DMSO). Unexpectedly, mTORC1 activity was significantly reduced in *Spry4*^G-KO^ tubules, with only about 4% of GFRα1^+^ cells positive for p-RPS6 (4.7-fold less) at BU-D10 (Figure 3C and 3D, G-KO, BU+DMSO). Furthermore, inhibition of ERK activity with PD treatment effectively increased the numbers of p-RPS6^+^ cells in *Spry4*^G-KO^ tubules to over 10% of GFRα1^+^ cells, comparable to that of *Spry4*^WT^ tubules after PD treatment (Figure 3C and 3D, BU+PD). No p-RPS6^+^GFRα1^+^ cells were detected in *Spry4*^G-KO^ or *Spry4*^WT^ mice receiving both low dose BU and mTORC1 inhibitor RAPA (Figure 3C and 3D, BU+RAPA).

Activation of mTORC1 has been reported to be essential for the regenerative response in spermatogonia^27^. Correspondingly, quantification of Ki67^+^ cells by whole-mount IF staining of *Spry4*^WT^ tubules revealed that the number of proliferating A_undiff_ spermatogonia was reduced from nearly 70% of GFRα1^+^ cells in mice undergoing normal regeneration (BU+DMSO) to about 20% of GFRα1^+^ cells in mice receiving RAPA treatment (BU+RAPA) (Figure 3E and 3F, WT). However, RAPA treatment did not affect proliferation of GFRα1^+^ cells in the tubules collected from *Spry4*^G-KO^ mice, further indicating that *Spry4* deletion inactivated mTORC1 in A_undiff_ spermatogonia at BU-D10 (Figure 3E and 3F, G-KO, BU+RAPA). Notably, inhibition of MAPK/ERK pathway activity with PD treatment, while partially suppressing spermatogonial proliferation in *Spry4*^WT^ tubules, significantly restored spermatogonia regeneration in *Spry4*^G-KO^ tubules, shown by an increased fraction of Ki67^+^GFRα1^+^ cells from 25% (BU+DMSO) to over 50% (BU+PD) (Figure 3E and 3F, G-KO). We also found that although BU treatment caused minimal damage in *Spry4*^G-KO^ tubules at BU-D10 (Figure 1D), PD treatment significantly impacted spermatogenesis with nearly 40% of tubules degenerated in both *Spry4*^G-KO^ and *Spry4*^WT^ mouse testes (Figure 3G and 3H, BU+PD), suggesting that an active MAPK/ERK pathway is required for spermatogonia differentiation.

### Hyper-activated MAPK/ERK pathway enhances spermatogonia proliferation in *Spry4*^G-KO^ testis shortly after damage

**Figure 4.**
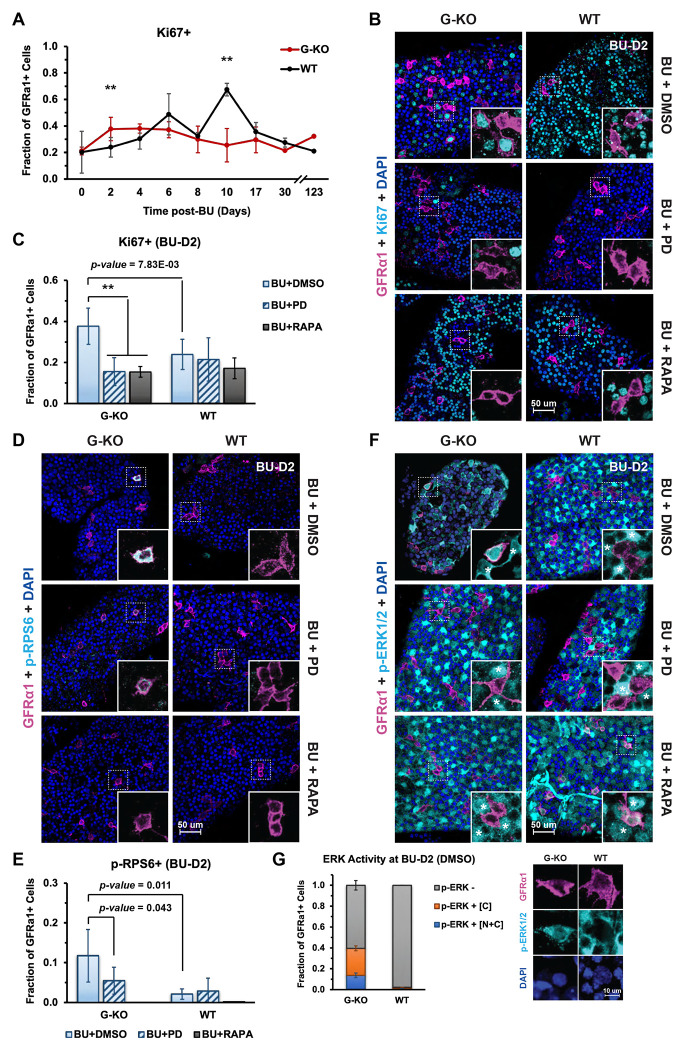
Hyper-activated ERK signaling enhances spermatogonia proliferation in *Spry4*^G-KO^ testis rapidly after damage (BU-D2). Adult *Spry4*^G-KO^ or *Spry4*^WT^ mice were treated with single low dose BU, and testes were collected at various time points post-BU as indicated. **(A)** Fraction of Ki67^+^ cells within GFRα1^+^ cells based on whole-mount IF. **, *p-value* < 0.01 (n>3 mice analyzed per condition per time point, n>100 GFRα1^+^ cells scored per animal). Mice were treated with single low dose BU together with DMSO (BU+DMSO), PD0325901 (BU+PD), or Rapamycin (BU+RAPA). Testes were collected 2 days post-BU (BU-D2) and analyzed by whole-mount IF staining for **(B)** Proliferation. Magenta, GFRα1; Cyan, Ki67; Blue, DAPI, **(D)** mTORC1 activity. Magenta, GFRα1; Cyan, p-RPS6; Blue, DAPI, or **(F)** ERK activity. Magenta, GFRα1; Cyan, p-ERK1/2; Blue, DAPI. Inset, higher magnification images of indicated regions without DAPI. Asterisks, somatic cells. GFRα1^+^ cells in whole-mount IF were counted for the fraction of **(C)** Ki67^+^ cells (**, *p-value* < 0.01) and **(E)** p-RPS6^+^ cells in the testes collected after indicated treatment, or **(G)** p-ERK1/2 enrichment within both nucleus and cytoplasm (p-ERK^+^ [N+C]), only in the cytoplasm (p-ERK^+^ [C]), or no detectable p-ERK1/2 (p-ERK^−^) at BU-D2 (BU+DMSO), with higher magnification images of GFRα1^+^ cells in *Spry4*^G-KO^ or *Spry4*^WT^ tubules shown on the left. n>3 mice analyzed per condition, n>100 GFRα1^+^ cells scored per animal. Data are mean (SD).

Our *Spry4*^G-KO^ mouse model at BU-D10 revealed the importance of appropriate ERK activity in regulating spermatogonia recovery during regeneration. To assess effects of germline-specific *Spry4* deletion during the initial phase of regeneration, we analyzed the proliferation of A_undiff_ spermatogonia by whole-mount IF staining of seminiferous tubules collected at multiple time points. Remarkably, shortly after damage (2 days post-BU, BU-D2) the *Spry4*^G-KO^ tubules contained more Ki67^+^GFRα1^+^ cells than *Spry4*^WT^ tubules (Figure 4A and 4B). Nearly 40% of GFRα1^+^ cells in the *Spry4*^G-KO^ tubules displayed strong Ki67 staining (Ki67^+^) until 6 days post-BU (BU-D6). By BU-D10, Ki67 decreased to a basal level (~20%) of GFRα1^+^ cells, where it was maintained long-term after recovering from damage (up to 123 days post-BU) (Figure 4A, G-KO). Conversely, most GFRα1^+^ cells remained quiescent in *Spry4*^WT^ tubules at BU-D2, then gradually turned active from 4 days post-BU (BU-D4) until reaching the peak for proliferation at BU-D10 (Figure 4A, WT). Inhibition of ERK or mTORC1 activity with PD or RAPA treatment did not affect GFRα1^+^ cell proliferation in *Spry4*^WT^ tubules at BU-D2 (Figure 4B and 4C, WT). However, both inhibitors reduced proliferation of the GFRα1^+^ cells in *Spry4*^G-KO^ tubules to similar levels that were comparable to those in *Spry4*^WT^ tubules (Figure 4B and 4C, G-KO). In addition, robust cytoplasmic p-RPS6 was identified in the GFRα1^+^ cells in *Spry4*^G-KO^ tubules; this was effectively blocked by PD or RAPA treatment (Figure 4D and 4E, G-KO). However, GFRα1^+^ cells in *Spry4*^WT^ tubules did not respond to either PD or RAPA treatment for enrichment of cytoplasmic p-RPS6 (Figure 4D and 4E, WT).

Notably, most tubules collected from *Spry4*^G-KO^ or *Spry4*^WT^ mice demonstrated profound p-ERK1/2 signaling in non-spermatogonial cells (MCAM^−^ or tdTomato^−^), revealing enhanced ERK activity within the somatic niche of germ cells shortly after damage (Figure 4F and S7, asterisks). p-ERK1/2 was identified within nearly 40% of GFRα1^+^ cells (26% mainly in cytoplasm and 14% within the entire cell) in *Spry4*^G-KO^ tubules but not in *Spry4*^WT^ tubules at BU-D2 (Figure 4F and 4G, BU+DMSO). Both PD and RAPA reduced the enrichment of p-ERK1/2 within GFRα1^+^ cells in *Spry4*^G-KO^ tubules, although p-ERK1/2 was detected within a small number of GFRα1^+^ cells in *Spry4*^WT^ tubules after RAPA treatment (Figure 4F, BU+PD and BU+RAPA). These data suggest that although low dose BU treatment does not induce a rapid regeneration response in *Spry4*^WT^ tubules, deletion of *Spry4* from spermatogonia results in hyperactive ERK right after damage. As such, stem cell proliferation is promoted rapidly yet transiently through ERK-mTORC1 signaling.

### SPRY4 is required for restoration of A_undiff_ spermatogonia and male fertility after damage

**Figure 5.**
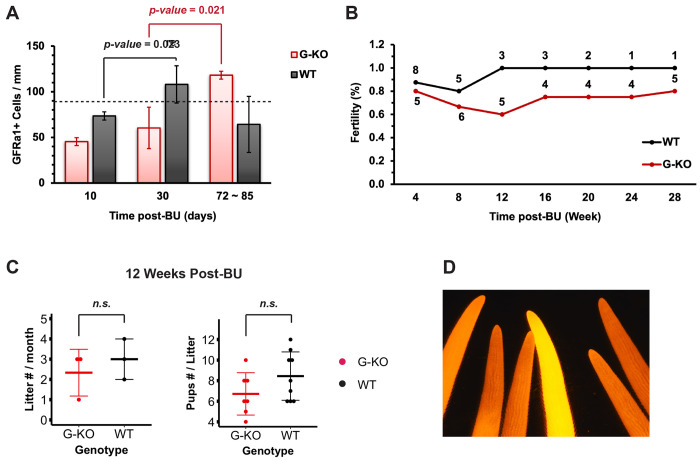
The recoveries of both spermatogonia pool and fertility rate are delayed in adult *Spry4*^G-KO^ males after damage. **(A)** Quantification of GFRα1^+^ cells per mm of seminiferous tubules collected from *Spry4*^G-KO^ or *Spry4*^WT^ males at different time points post-BU. Dashed line, GFRα1^+^ cells per mm in adult *Spry4*^WT^ males at steady state. Data are mean (SD), n>3 mice analyzed per genotype. **(B)** Fertility rate of adult *Spry4*^G-KO^ or *Spry4*^WT^ males post-BU. Number in the linear graph, number of mice tested at the indicated time point. **(C)** Number of litters (left) and offspring pups (right) produced by *Spry4*^G-KO^ or *Spry4*^WT^ males 12 weeks post-BU. **(D)** Red fluorescence (tdTomato) image from the tails of one litter of pups produced by a *Spry4*^G-KO^ male 28 weeks post-BU.

To explore the consequences of germline-specific *Spry4* deletion on spermatogonia regeneration in long-term, we applied whole-mount IF to analyze GFRα1^+^ cells at multiple time points after BU treatment. At 30 days post-BU (BU-D30), the GFRα1^+^ cell density within *Spry4*^WT^ tubules recovered to steady state (Figure 5A). However, similar to the *Spry4*^G-KO^ tubules collected at BU-D10 (Figure 1E and 1F), GFRα1^+^ cells remained sparsely located within the *Spry4*^G-KO^ tubules at BU-D30, with the corresponding GFRα1^+^ cell densities lower than those collected from *Spry4*^WT^ tubules (Figure 5A, S8A and S8B). Furthermore, most GFRα1^+^ cells (~80%) in the *Spry4*^G-KO^ tubules persisted as the most primitive A_s_ spermatogonia, at a significantly higher rate than those identified in the *Spry4*^WT^ tubules at BU-D30 (Figure S8C). Conversely, the fractions of A_al_ spermatogonia were reduced in *Spry4*^G-KO^ tubules (Figure S8C). Notably, the number of GFRα1^+^ cells doubled within two months in *Spry4*^G-KO^ tubules after BU-D30, suggesting a delayed spermatogonia regeneration with *Spry4* deletion (Figure 5A). We also found that although *Spry4*^WT^ mice became completely fertile by 12 weeks post-BU (6-month-old), *Spry4*^G-KO^ mice retrieved fertility much slower, with one male remained sterile within the entire breeding test period (Figure 5B). All the fertile *Spry4*^G-KO^ males recovered from damage were capable of efficient breeding by producing similar number of litters and offspring pups as *Spry4*^WT^ males (Figure 5C). And strong tdTomato signal was observed in every pup produced by *Spry4*^G-KO^ males, revealing efficient GFRα1-Cre^ERT2^-induced recombination within the mature *Spry4*^G-KO^ spermatozoa recovered from regeneration (Figure 5D).

## Discussion

Life-long spermatogenesis requires coordinated self-renewal and balanced differentiation of germ cells to prevent exhaustion of SSCs while maintaining continual germ cell generation. While the molecular and cellular pathways orchestrating spermatogenesis are well documented, the mechanism(s) by which the SSC compartment responds to physiological stress to sustain its repertoire of regenerating and differentiating germ cells is less well defined. We have previously shown that MAPK/ERK-dependent negative feedback regulator SPRY4 enables mouse SSCs to respond to growth factor signaling to preserve stem cell activity in culture. Indeed, *Spry4* serves as a marker for adult SSCs *in vivo* by uniquely expressing in a subset of A_undiff_ spermatogonia but not in differentiating germ cells in adult mouse testis^30^. These observations raised the possibility that SPRY4 might perform as a rheostat balancing coordinated germ cell recovery without exhausting the vulnerable SSC compartment. Thus, to decipher the SPRY4 function in the regeneration of SSCs *in vivo*, we employed single low dose BU treatment to deplete most SSCs and rapidly dividing spermatogonia from testis and follow the recovery of the remaining stem cells over time after injury. We demonstrate that in the *Spry4*^G-KO^ mice, loss of *Spry4* in the spermatogonia enabled us to define a cell-autonomous role for SPRY4-ERK signaling in securing rapid germline recovery and restoring long-term fertility in adult mouse testis. Thus, SPRY4 is a novel molecular hub balancing SSC homeostasis under stress to meet the demand for continual generation of mature germ cells.

**Figure 6.**
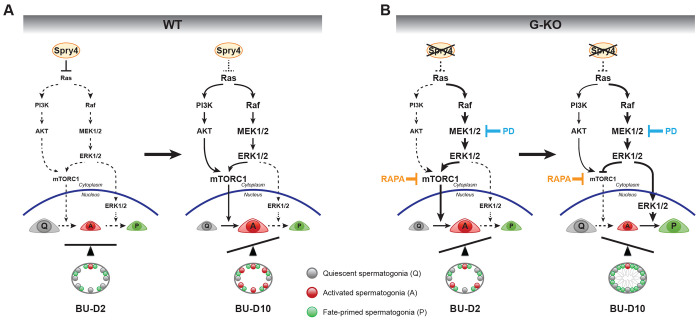
Model illustrating SPRY4-ERK function during spermatogonia recovery and regeneration in BU-damaged mouse testis. **(A)** Wild-type spermatogonia achieve regeneration through a SPRY4-controlled signaling transition. At BU-D2, SPRY4 imposes repression to RTK signaling, inactivating both MAPK/ERK pathway and mTORC1, and poises the surviving A_undiff_ spermatogonia in quiescent state. By BU-D10, MAPK/ERK pathway is released to activate ERK1/2 in the cytoplasm of A_undiff_ spermatogonia, which then function as a major activator of mTORC1. Activated mTORC1 promotes A_undiff_ spermatogonia transition from quiescent to cell-cycle-activated phase (proliferation) and initiates regeneration. **(B)** In *Spry4*^G-KO^ spermatogonia, both ERK1/2 and mTORC1 are activated in the cytoplasm of the surviving A_undiff_ spermatogonia immediately after damage (BU-D2), resulting in rapid proliferation in response to injury. By BU-D10, hyperactivated ERK1/2 locates to the nucleus of A_undiff_ spermatogonia and promotes fate commitment, suspending A_undiff_ spermatogonia proliferation with inactivated mTORC1. Q, quiescent A_undiff_ spermatogonia; A, activated A_undiff_ spermatogonia; P, fate-primed spermatogonia; PD, PD0325901; RAPA, Rapamycin.

In the seminiferous tubules of wild-type (*Spry4*^WT^) mouse testis, SPRY4 represses RTK-dependent signaling within the surviving A_undiff_ spermatogonia rapidly after damage (BU-D2), which inactivates both MAPK/ERK and mTORC1 signaling and pauses A_undiff_ spermatogonia in a quiescent state. By BU-D10, ERK1/2 is activated through the MAPK/ERK pathway and enriched in the cytoplasm of A_undiff_ spermatogonia, which cooperates with other signaling pathways (mainly PI3K/AKT) to activate mTORC1 and induce robust proliferation in A_undiff_ spermatogonia (Figure 6A). Deletion of the *Spry4* gene from spermatogonia unleashes RTK-dependent signaling from repression, which activates cytoplasmic ERK1/2 and mTORC1 quickly after damage and induces proliferation in A_undiff_ spermatogonia, resulting in rapid initiation of germ cell regeneration at BU-D2. However, hyperactivated ERK1/2 translocates to the nucleus of A_undiff_ spermatogonia by BU-D10, triggering spermatogonia differentiation while simultaneously blocking mTORC1-supported spermatogonia proliferation (Figure 6B). Deleting the *Spry4* gene from spermatogonia delays the recovery of A_undiff_ spermatogonia pool after injury and reduces long-term fertility rate in young adult males.

Our study sets forth the notion that during stress spermatogenesis, SPRY4-ERK signaling in the surviving stem cells executes a regeneration program to ensure appropriate germline recovery without exhausting SSCs. SPRY4 is dispensable for spermatogenesis in young adult mice but acts as a critical cell-intrinsic molecular rheostat of the MAPK/ERK pathway to modulate the progression of balanced germline regeneration. SPRY4 pauses the regenerative responses in A_undiff_ spermatogonia immediately after damage by diverting MAPK/ERK pathway to activate mTORC1, which promotes spermatogonia proliferation and replenishing mature germ cells. During the progression of regeneration, SPRY4 is still required to appropriately balance stem cell proliferation with differentiation through regulating ERK1/2 activity. Loss of *Spry4* in A_undiff_ spermatogonia accelerates the regeneration response to BU damage, with hyperactivated ERK1/2 further promoting spermatogonia differentiation at the expense of self-renewal. While mTORC1 signaling may be necessary for supporting spermatogonia regeneration, it is insufficient. Although modest ERK activity in the cytoplasm may promote stem cell regeneration through mTORC1 signaling, *Spry4* deletion-induced ERK hyper-activation and nuclear localization significantly inhibits mTORC1 activation and spermatogonia recovery from damage while promoting spermatogonia differentiation, which can be partially rescued by pharmacologically inhibiting the MAPK/ERK pathway.

We identified several genes that are potentially targeted by SPRY4-ERK signaling in the regenerative A_undiff_ spermatogonia. Notably, *Id1* was down-regulated in *Spry4*^G-KO^ spermatogonia at BU-D10. ID proteins serve as dominant-negative inhibitors of E-box-binding transcription factors (E proteins) that activate cell fate specification and differentiation in stem cells^35^. A study with serial bone marrow transplantation assays revealed that deleting *Id1* gene increases self-renewal and decreases proliferation in mouse hematopoietic stem cells (HSCs)^39^. In both embryonic and adult mouse brains, ID1 and other ID family proteins have been shown to preserve neural stem cell (NSC) self-renewal capacity by preventing premature differentiation^40,41^. In adult mice, VEGFR2-ID1-mediated inductive angiogenesis in liver sinusoidal endothelial cells (LSECs) establishes an inductive vascular niche to initiate liver regeneration^42^. However, ID1 function in mammalian SSCs remains unknown. Notably, ID4 selectively marks A_s_ spermatogonia (SSCs) and is suggested to regulate SSC self-renewal in mice^43–45^, and a recent study suggests that ID family transcriptional regulators are functionally redundant in SSCs^46^. In our study of regenerative SSCs at BU-D10, down-regulated *Id1* gene expression in *Spry4*^G-KO^ A_undiff_ spermatogonia corresponds with more quiescent SSCs at BU-D10, suggesting the role of ID1 in supporting germline regeneration under genotoxic stress. The expression of *Id1* in *Spry4*^G-KO^ A_undiff_ spermatogonia can be restored by inhibition of the MAPK/ERK pathway with PD treatment, which further demonstrates that the SPRY4-ERK signaling efficiently regulates *Id1* gene *in vivo*. ID proteins may serve as executors of SPRY4-ERK signaling during regeneration. It remains to be determined whether ID1 is specifically required for SSC regeneration, and whether the expression of *Id1* and other ID family members are directly regulated by SPRY4-ERK signaling after locating p-ERK1/2 into the nucleus.

Among the genes upregulated in regenerative *Spry4*^G-KO^ spermatogonia, *Cxcl12* plays a critical role in mammalian stem cell development primarily through binding to its cognate receptor CXCR4^47^. Appropriate CXCL12–CXCR4 signaling is essential to maintain the SSC population in culture and stem cell homing/colonization capacity in transplantation. Inhibition of CXCL12–CXCR4 signaling in adult mouse testis impairs SSC maintenance, leading to loss of the germline^48^. CXCL12 is also required for the maintenance of other adult stem cells (i.e., HSC^49^, NSC^50^, etc.), disease development^51^, or tissue repair^52^. CXCL12 is significantly upregulated in *Spry4*^G-KO^ A_undiff_ spermatogonia but reduced with PD treatment at BU-D10, reflecting a SPRY4-ERK signaling induced transition from differentiation-primed state to self-renewal state in regenerative spermatogonia.

As one of the major negative-feedback regulators of RTK-dependent signaling pathways, SPRY4 activity can be tightly controlled through its intracellular localization, post-translational modification, or binding with cofactors including other Sprouty family isoforms^53^. Our study in wild-type A_undiff_ spermatogonia revealed that SSCs activate their regenerative responses through RTK-dependent PI3K/AKT and MAPK/ERK pathways at least two days after chemotherapy-induced damage. Deletion of the *Spry4* gene in spermatogonia augmented SSC regeneration. Given that *Spry4* is exclusively expressed in A_undiff_ spermatogonia within the adult mouse germ cells^30^, which presumably inhibits RTK-dependent signaling pathways in SSCs at steady state and immediately after injury (within 2 days of BU treatment), pausing SPRY4 activity in SSCs would be required to initiate regeneration in wild-type mouse. It has been reported that a heterooligomer formed between SPRY4 and SPRY1 exhibits the most potent inhibitory effect on ERK activation in cultured mouse or human cells^54^, suggesting that the activities of other Sprouty family isoforms in A_undiff_ spermatogonia may also facilitate SPRY4 function during regeneration. Our study identified the role of SSC-intrinsic SPRY4 in responding to damage, further investigation about how to regulate the activities of SPRY4 and other Sprouty family isoforms in germ cells will be needed. Considering the essential role of stem cell microenvironment in maintaining and regulating stem cell function, somatic cells that constitute SSC niche (i.e., Sertoli, Leydig, peritubular myoid and testicular endothelial cells, etc.) may also modulate SPRY4 activities within SSCs and promote regeneration after injury through multiple mechanisms. Indeed, Sertoli cells have been reported to periodically activate ERK1/2 and produce GDNF to support SSC self-renewal at selected stage of seminiferous cycle^21^. Specialized testicular endothelial cells have also been identified as a critical population in the germline stem cell niche by producing GDNF and other factors to support human and mouse SSCs in culture^55^.

Both somatic stem cells and SSCs are vulnerable to DNA damage induced by chemotherapy, resulting in severe side effects or long-term tissue dysfunction (i.e. infertility) in cancer patients post-treatment^56^. Specifically, there are no means to preserve the fertility of prepubertal male patients, who are not yet producing mature sperms and therefore unable to benefit from standard sperm banking^57^. Elucidation of the signaling pathways and regulatory networks that control stem cell self-renewal, proliferation, and differentiation in adolescent and adult organs is essential to develop targeted therapies, improve tissue repair, and advance regenerative medicine. By dissecting the key transition steps in recovering SSCs, our study identifies SPRY4-ERK signaling as the first critical checkpoint regulator that modulates germline-specific damage response to restore male reproduction in adult mammals. Our results reveal a novel negative feedback regulator-dominant mechanism in orchestrating cell-intrinsic signaling to coordinate adult stem cell recovery and long-term homeostasis, shedding a light on improving tissue regeneration after drug induced injury and preserving human fertility from environmental stresses.

## Materials and methods

### Ethics Statement

This study was approved by the Weill Cornell Medical College IACUC (#2010-0028). For euthanasia, mice were exposed to CO_2_ followed by cervical dislocation.

### Mouse Maintenance and Treatment

The *Gfra1-CreER*^*T2*^ mice were a gift from Dr. Sanjay Jain^31,58^. *Rosa26-lox-stop-lox(LSL)-tdTomato* reporter mice were obtained from Jackson Laboratory (strain #: 007914)^59^. The *Spry4*^*flox*^ mice were a gift from Dr. Ophir Klein^60^. The genotype of the newborn mice was determined by PCR with primer listed in Supplementary Table 1. For gene deletion, tamoxifen (HY-13757A, MedChemExpress) was administered to adult mice (2- to 4-month-old) by intraperitoneal (IP) injection at a dose of 100 mg/kg of body weight once every 24 h for 4 consecutive days per week for 2 weeks total. To induce regeneration, busulfan (HY-B0245, MedChemExpress) was administered to mice by IP injection at a single dose of 10 mg/kg of body weight. BU-treated cohorts were treated daily by IP injection starting from 3 days post-BU for 7 consecutive days with PD0325901 (HY-10254, MedChemExpress, 2.5 mg/kg), Rapamycin (HY-10219, MedChemExpress, 4 mg/kg) or vehicle (DMSO).

### Sperm counting

After euthanizing the mice, one cauda epididymide was dissected from each mouse and thoroughly minced in a 1.5 ml microcentrifuge tube containing 1 ml PBS. The sample was left in room temperature for 15 min to allow the sperms to swim out. The sperm suspension was diluted 10 folds with PBS and counted on a hemocytometer. Sperm counts were obtained twice and recorded as the average of the two counts.

### Hematoxylin and eosin (H&E)

Tissues were fixed with 4% paraformaldehyde/PBS overnight at 4 °C, washed and immersed in PBS. H&E staining was performed at center for translational pathology at Weill Cornell Medicine following standard methods. Images were captured with an ECHO Revolve microscope and tissue architecture was scored blindly.

### Whole-mount Immunofluorescence (IF)

For whole-mount IF, seminiferous tubules were teased apart from freshly collected detunicated testes and rinsed in PBS. Tubules were fixed with 4% PFA (158127, Sigma-Aldrich) overnight at 4°C and then washed with PBS for three times. The tubules were incubated with the basic optical clearing solution consists of 150mM KCl, 20% DMSO, 0.5% triton, 0.5% NaN3, and 10mM Tris-HCl for 20 min at 37°C to decolorize the tissue and reduce autofluorescence^61^. Tubules were then blocked with 1 % normal donkey serum albumin (A1470,Sigma-Aldrich) for 1 hr at room temperature in PBS before incubation with 1 μg/ml goat anti-GFRα1 (AF560, R&D Systems), 1 μg/ml rat anti-MCAM antibody (134702, BioLegend), 1:250 rabbit anti-phospho-p44/42 MAPK (Erk1/2) antibody (4370S, Cell Signaling), 1 ug/ml Ki67 (ab15580, Abcam), or 1:500 rabbit anti-pRPS6 (9101S, Cell Signaling), overnight at 4°C in a nutating mixer at 4°C. Detection of primary antibodies were performed using 1 μg/ml Alexa-647 conjugated Anti-Rabbit IgG (711-065-152, Jackson ImmunoResearch), Alexa-488 conjugated Anti-Goat IgG (705-545-147, Jackson ImmunoResearch), anti-rat IgG 568 (A-11077, Invitrogen), or anti-rabbit IgG 647 (711-605-152, Jackson ImmunoResearch) for 60 min at room temperature. Note, all the incubation were done in the nutating mixer to ensure proper penetration of solutions. The tubules were mounted in PBS with DAPI in a 35-mm glass-bottomed dish (Part No: P35G-0-14-C, MatTek) and top with a glass cover slip. Image analysis was performed on a Zeiss LSM800 confocal microscope.

### IF image analysis

To quantify spermatogonia in adult mouse testis, the seminiferous tubules were examined by whole-mount IF staining for GFRα1. The density and the fraction of A_s_, A_pr_, and A_al_ spermatogonia were calculated by scoring n>100 GFRα1^+^ cells within over 5 mm of seminiferous tubules per animal. 3 – 5 animals were analyzed per condition.

### Fluorescence-activated cell sorting (FACS)

To isolate spermatogonia, testes were collected from adult mice 10 days after recovering from BU treatment (BU-D10). Single-cell suspensions were generated from testes by enzymatic digestion as described before^62^. Briefly, seminiferous tubules were collected from detunicated testes and minced on ice. The tissue was enzymatically dissociated with agitation for 30 min at 37°C in a buffer containing 0.017% trypsin (Cellgro), 17mM EDTA (Cellgro), 0.03% collagenase (Sigma-Aldrich) and DNase I (100 μg/ml; Roche). For fluorescence-activated cell sorting (FACS), spermatogonia were firstly enriched for MCAM/CD146^+^, a marker of SSCs and spermatogonia^32,33^, via mouse CD146 (LSEC) MicroBeads (Miltenyi Biotec, 130-092-007), then proceeded with antibody staining with Alexa Fluor 647 anti-MCAM antibody (clone ME-9F1, 134718, BioLegend), Alexa Fluor 488 anti-c-Kit (CD117) antibody (clone 2B8, 105816, BioLegend), and PE/Cyanine7 anti-CD31 antibody (clone WM59, 303118, BioLegend) each at 10 μg/ml for 30 min at 4°C. After bead enrichment, about 1 – 2 x10^6^ testicular cells were collected for FACS. Cell suspensions with immunocomplexes were subject to FACS with a BD FACS Aria II instrument (Beckman Coulter, USA). DAPI (D1306, Thermo Fisher Scientific) was used for live/dead cell discrimination. After exclusion of doublets and DAPI-positive cells, as well as testicular endothelial cells (CD31^+^), the undifferentiated germ cell population (MCAM^High^c-Kit^−^, including GFRα1^+^ A_undiff_ spermatogonia) were gated and collected.

### FACS analysis

FACS results were analyzed with Flowjo 9.3.2. SSC Differentiation Index (SDI) was calculated by: SDI = Fraction of MCAM^Low^CD31^−^ cells / Fraction of MCAM^High^c-Kit^−^CD31^−^ cells

### RNA extraction

Total RNA was extracted from fresh spermatogonia isolated by FACS using PicoPure RNA isolation kit (KIT0204, Applied Biosystems) according to the manufacturer’s instructions. Residual genomic DNA was removed by on-column DNA digestion (79254, Qiagen).

### Library construction and sequencing

The stranded mRNA sequence libraries were generated using the KAPA mRNA HyperPrep Kit (Kapa Biosystems, KK8580) according to the manufacturer’s instructions. Sequencing service was performed on an Illumina NovaSeq X plus sequencer according to the standard Illumina protocol.

### Quantitative reverse transcription polymerase chain reaction (qRT-PCR)

qRT-PCR was performed in triplicate in a LightCycler 480II Real-Time System (Roche Molecular Systems) using Luna Universal qPCR Master Mix (M3003E, NEB). Primers were validated for use based on efficiency and inspection of melting curves. For quantification, each technical triplicate was normalized to endogenous *Tbp* and relative transcript expression was calculated using the comparative CT method (2^−ΔΔ^*Ct* method) using the average value of the technical triplicates of the control condition as the reference value. Data presented correspond to *Tbp* normalization. The qRT-PCR primers are listed in Supplementary Table 2.

### RNA-seq Data Processing and Analysis

Sequencing reads were de-multiplexed, checked for quality (FastQC), and trimmed/filtered when appropriate. The resultant high quality reads were mapped (STAR v2.7.10a) to the transcriptome sequence reference of the GENCODE mm10 GRCm39 version 107 build. Gene counts and transcript abundance measures (FPKM values) were quantified using the R package edgeR (v4.0.14). Gene expression was reported as log2 transformed FPKM value. With Limma R package and expression profiles generated in our labs, lists of the differentially expressed genes in the unpair-wise comparisons between gene deletion and control samples were generated with at least one-fold (log2) change and statistical significances (*p-value* < 0.05). At least three biological replicates from each sample were analyzed.

### Statistical analyses

Results are presented as means ± SD. At least three biological replicates and three technical replicates were performed for each experiment unless otherwise indicated in the text. Results were considered significant at *p* value <0.05. R (version 4.4.1) was used for statistical analyses and generating graphs.

## Supplementary Material

Supplement 1 **Supplementary Figure 1. Fluorescent reporter in *Spry4*^G-KO^ testis labels germ cells of all stages.** Testes collected from adult *Spry4*^G-KO^ (G-KO) mice 6 weeks post-tamoxifen treatment were analyzed by fluorescent microscopy for **(A)** tdTomato (tdT) expression in whole-mount seminiferous tubules. Red, tdTomato; Blue, DAPI. Representative whole-mount IF images illustrating tdTomato^+^ populations (Red) in tubules collected from *Spry4*^G-KO^ mice. **(B)** Light blue, VASA; **(C)** Magenta, GFRα1; Green, MCAM; **(D)** Grey, Vimentin; Green, MCAM; Blue, DAPI. Inset, higher magnification images of indicated regions without DAPI or tdT.
**Supplementary Figure 2. Characterization of germline-specific *Spry4* deletion in adult mouse testis at steady state.** Testes collected from adult (within 4 months of age) *Spry4*^G-KO^ (G-KO) or *Spry4*^WT^ (WT) mice 6 weeks post-tamoxifen treatment were compared for **(A)** testis to body weight ratio and **(B)** sperm count. *n.s.*, not significant (*p-value* >= 0.05). Data are mean (SD), n>5 mice analyzed per genotype. **(C)** H&E–stained histological cross-sections of testis (up), caput and cauda epididymis (down).
**Supplementary Figure 3. H&E–stained histological cross-sections of mouse testes and epididymides.** Organs collected from adult (4-month-old) *Spry4*^G-KO^ (G-KO) or *Spry4*^WT^ (WT) mice treated with **(A)** single low dose BU (10 mg/kg) or **(B)** DMSO. Testis (up) and epididymis (down) were collected 10 days after treatment. These images reveal the lower magnification areas from Fig 1D.
**Supplementary Figure 4. FACS strategy for isolating spermatogonia. (A)** A_undiff_ spermatogonia (MCAM^High^c-Kit^−^CD31^−^) and A_diff_ spermatogonia (MCAM^Low^CD31^−^) isolated from mouse CD146 MicroBeads-enriched testicular cells. Percentages of cells within gates are indicated. **(B)** tdTomato+ cells within MCAM^High^c-Kit^−^ cells isolated from *Spry4*^G-KO^ mouse testes.
**Supplementary Figure 5. qRT-PCR analysis of selected genes.** *, *p-value* < 0.05; **, *p-value* < 0.01.
**Supplementary Figure 6. ERK activity within the seminiferous tubules during regeneration (BU-D10).** Adult mice were treated with single low dose BU and then daily with DMSO (BU+DMSO), PD0325901 (BU+PD), or Rapamycin (BU+RAPA) starting from day 3 post-BU. Testes were collected for analysis at BU-D10 by whole-mount IF staining for ERK activity in the seminiferous tubules collected from **(A)** *Spry4*^WT^ mice. Red, MCAM; Cyan, p-ERK1/2; Blue, DAPI, or **(B)** *Spry4*^G-KO^ mice. Red, tdTomato; Grey, GFRα1; Cyan, p-ERK1/2; Blue, DAPI. Inset, higher magnification images of indicated regions without DAPI. Asterisks, somatic cells with p-ERK1/2.
**Supplementary Figure 7. ERK activity within the seminiferous tubules quickly after damage (BU-D2).** Adult mice were treated with single low dose BU and then daily with DMSO (BU+DMSO), PD0325901 (BU+PD), or Rapamycin (BU+RAPA) starting from day 0 post-BU. Testes were collected for analysis at BU-D2 by whole-mount IF staining for ERK activity in the seminiferous tubules collected from **(A)** *Spry4*^WT^ mice. Red, MCAM; Cyan, p-ERK1/2; Blue, DAPI, or **(B)** *Spry4*^G-KO^ mice. Red, tdTomato; Grey, GFRα1; Cyan, p-ERK1/2; Blue, DAPI. Inset, higher magnification images of indicated regions without DAPI. Asterisks, somatic cells with p-ERK1/2.
**Supplementary Figure 8. Spermatogonia remain reduced in adult *Spry4*^G-KO^ mice 30 days after damage (BU-D30). (A)** Testes collected from adult G-KO or WT mice at 30 days post-BU were analyzed by whole-mount IF for GFRα1^+^ populations. Higher magnification images of indicated regions are shown on the right. Magenta, GFRα1; Blue, DAPI. Asterisks, A_s_ spermatogonia; Brackets, A_pr_ spermatogonia; Dashed lines, A_al_ spermatogonia (4 – 8 cells). **(B)** Quantification of GFRα1^+^ cells per mm of seminiferous tubules. **(C)** Fraction of A_s_, A_pr_, and A_al_ spermatogonia based on whole-mount IF analysis of GFRα1^+^ clones. Number in round brackets indicates cell numbers per GFRα1^+^ clone. Data are mean (SD), n>3 mice analyzed per genotype.
Supplementary Table 1. Primer sequences for genotyping
Supplementary Table 2. Primer sequences for qRT-PCR

## Data Availability

The data underlying this article are available in the article, in its online supplementary material, and at the following link: (https://tbd).
