# Supplementary material for: Restoration of Spermatogenesis is Dependent on Activation of a SPRY4-ERK Checkpoint Following Germline Stem Cell Damage
Source: bioRxiv. 2025 Oct 14:2025.10.12.681919. Preprint. [Version 1] doi: 10.1101/2025.10.12.681919 (PMC12632827; doi:10.1101/2025.10.12.681919)
Supplement: Supplement 1 — Supplementary Figure 1. Fluorescent reporter in Spry4G-KO testis labels germ cells of all stages. Testes collected from adult Spry4G-KO (G-KO) mice 6 weeks post-tamoxifen treatment were analyzed by fluorescent microscopy for (A) tdTomato (tdT) expression in whole-mount seminiferous tubules. Red, tdTomato; Blue, DAPI. Representative whole-mount IF images illustrating tdTomato+ populations (Red) in tubules collected from Spry4G-KO mice. (B) Light blue, VASA; (C) Magenta, GFRα1; Green, MCAM; (D) Grey, Vimentin; Green, MCAM; Blue, DAPI. Inset, higher magnification images of indicated regions without DAPI or tdT. Supplementary Figure 2. Characterization of germline-specific Spry4 deletion in adult mouse testis at steady state. Testes collected from adult (within 4 months of age) Spry4G-KO (G-KO) or Spry4WT (WT) mice 6 weeks post-tamoxifen treatment were compared for (A) testis to body weight ratio and (B) sperm count. n.s., not significant (p-value >= 0.05). Data are mean (SD), n>5 mice analyzed per genotype. (C) H&E–stained histological cross-sections of testis (up), caput and cauda epididymis (down). Supplementary Figure 3. H&E–stained histological cross-sections of mouse testes and epididymides. Organs collected from adult (4-month-old) Spry4G-KO (G-KO) or Spry4WT (WT) mice treated with (A) single low dose BU (10 mg/kg) or (B) DMSO. Testis (up) and epididymis (down) were collected 10 days after treatment. These images reveal the lower magnification areas from Fig 1D. Supplementary Figure 4. FACS strategy for isolating spermatogonia. (A) Aundiff spermatogonia (MCAMHighc-Kit−CD31−) and Adiff spermatogonia (MCAMLowCD31−) isolated from mouse CD146 MicroBeads-enriched testicular cells. Percentages of cells within gates are indicated. (B) tdTomato+ cells within MCAMHighc-Kit− cells isolated from Spry4G-KO mouse testes. Supplementary Figure 5. qRT-PCR analysis of selected genes. *, p-value < 0.05; **, p-value < 0.01. Supplementary Figure 6. ERK activity within the seminifero [file media-1.pdf]

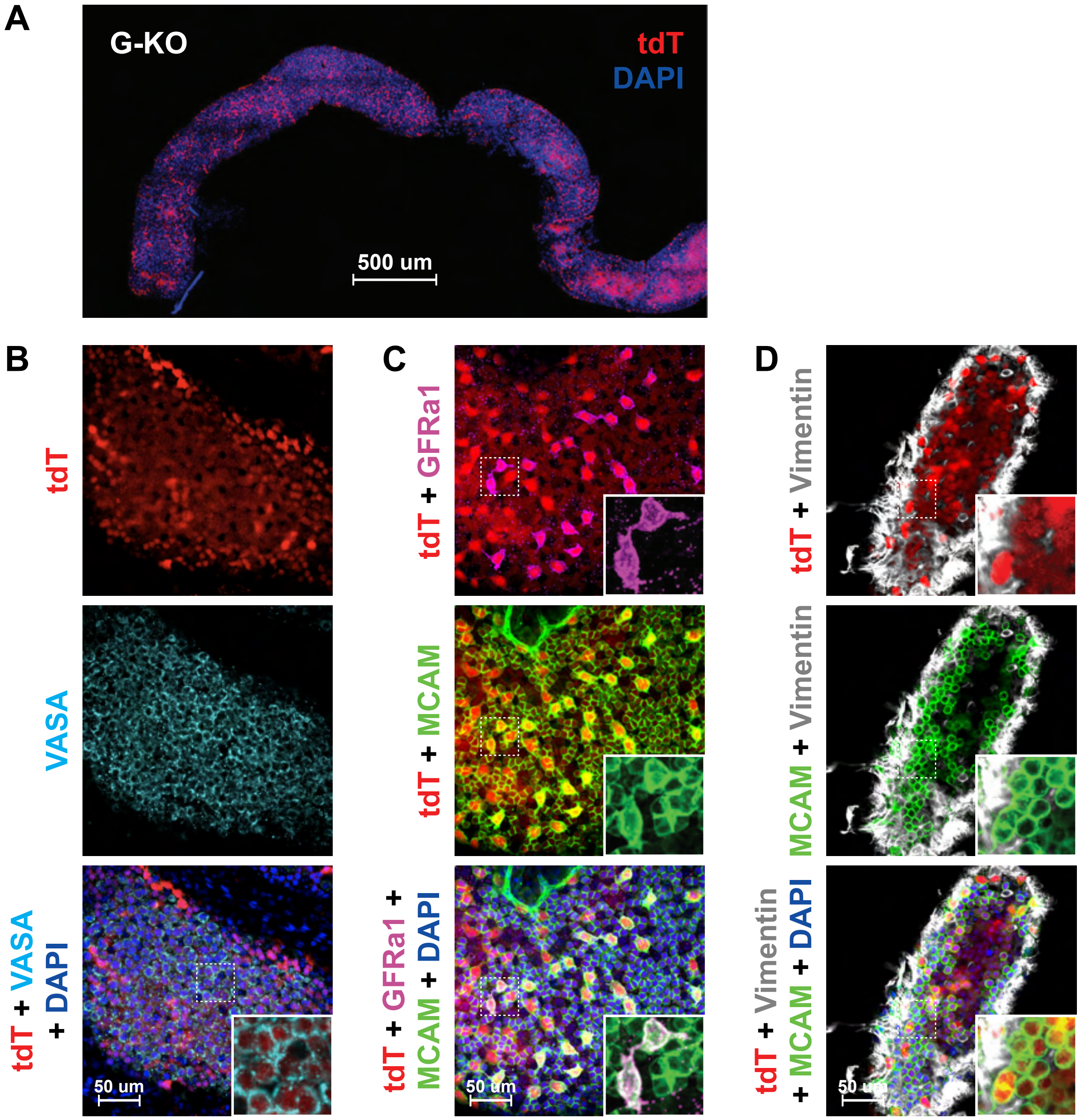

Supplementary Figure 1. Fluorescent reporter in the *Spry4*$^{\text{G-KO}}$ testis labels germ cells of all stages.

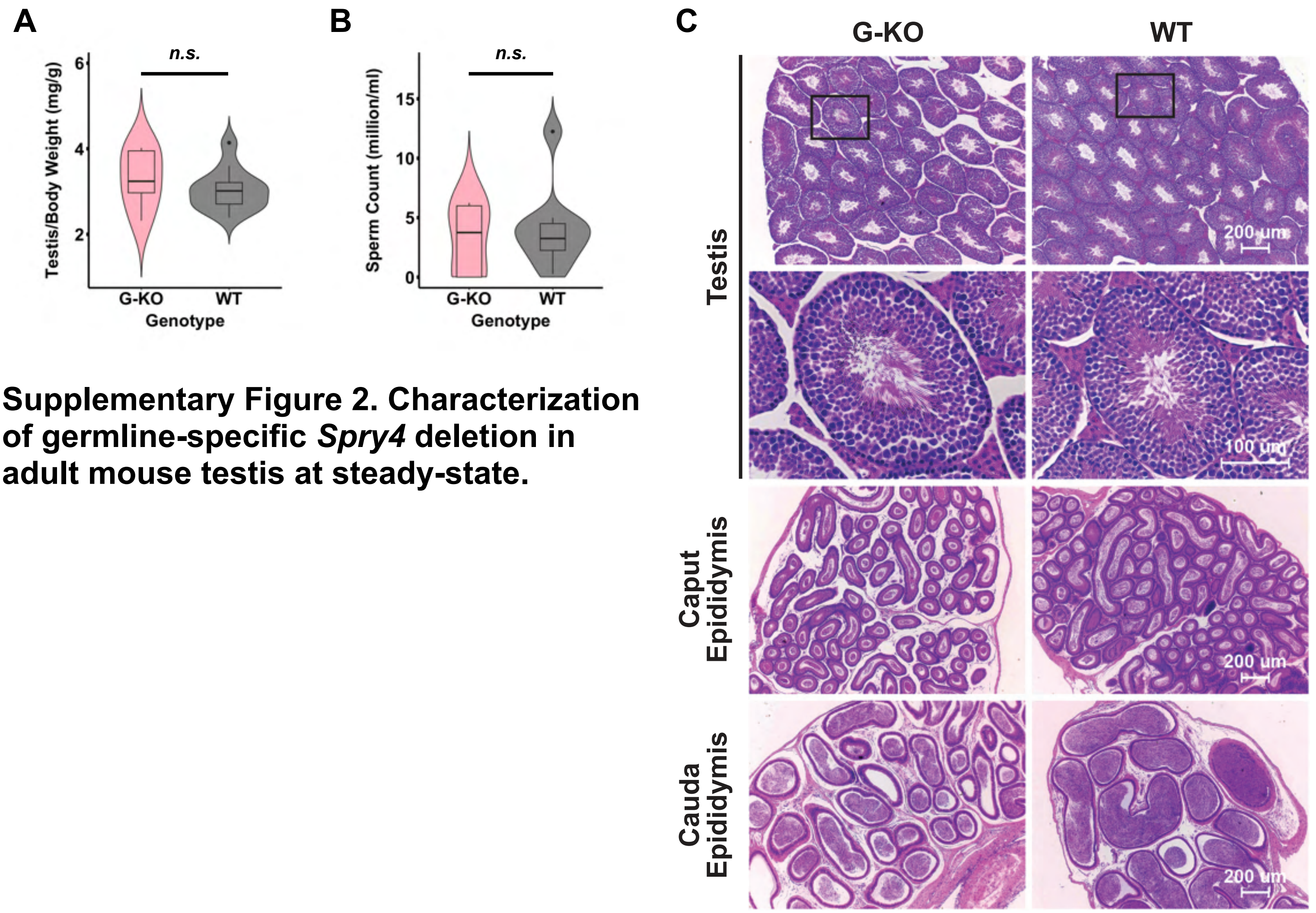

Supplementary Figure 2. Characterization of germline-specific *Spry4* deletion in adult mouse testis at steady-state.

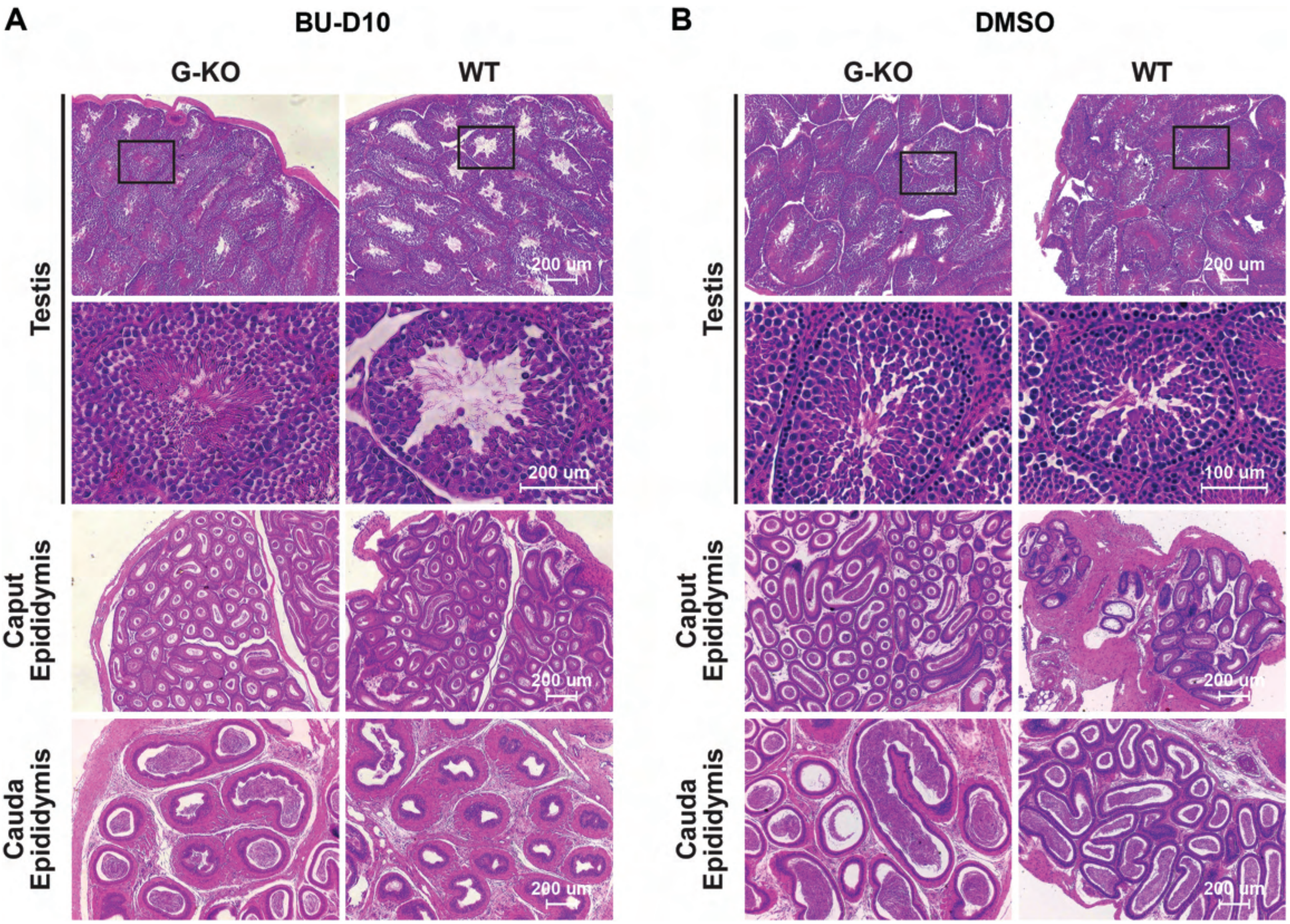

Supplementary Figure 3. H&E-stained histological cross-sections of mouse testes and epididymides.

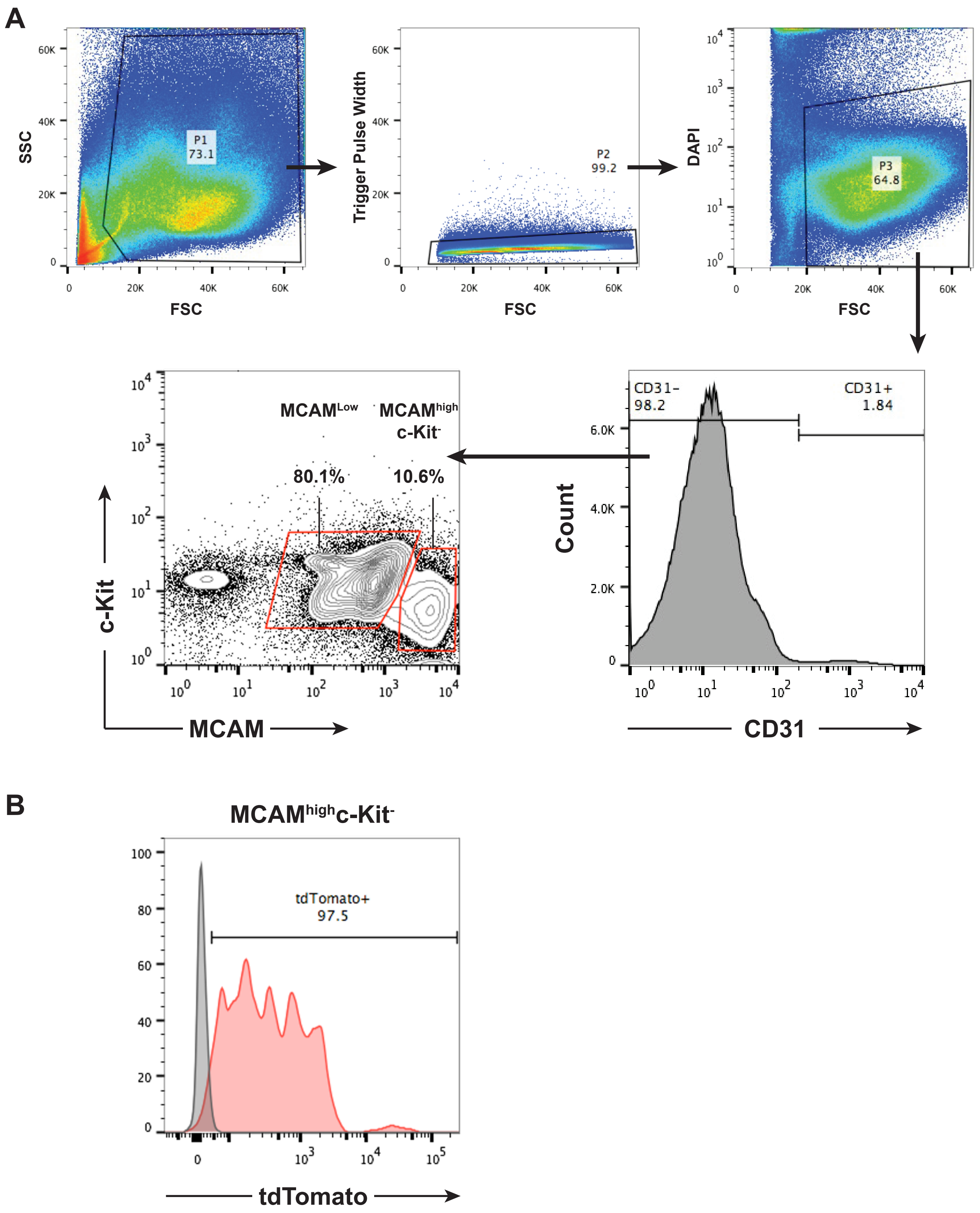

Supplementary Figure 4. FACS strategy for isolating spermatogonia.

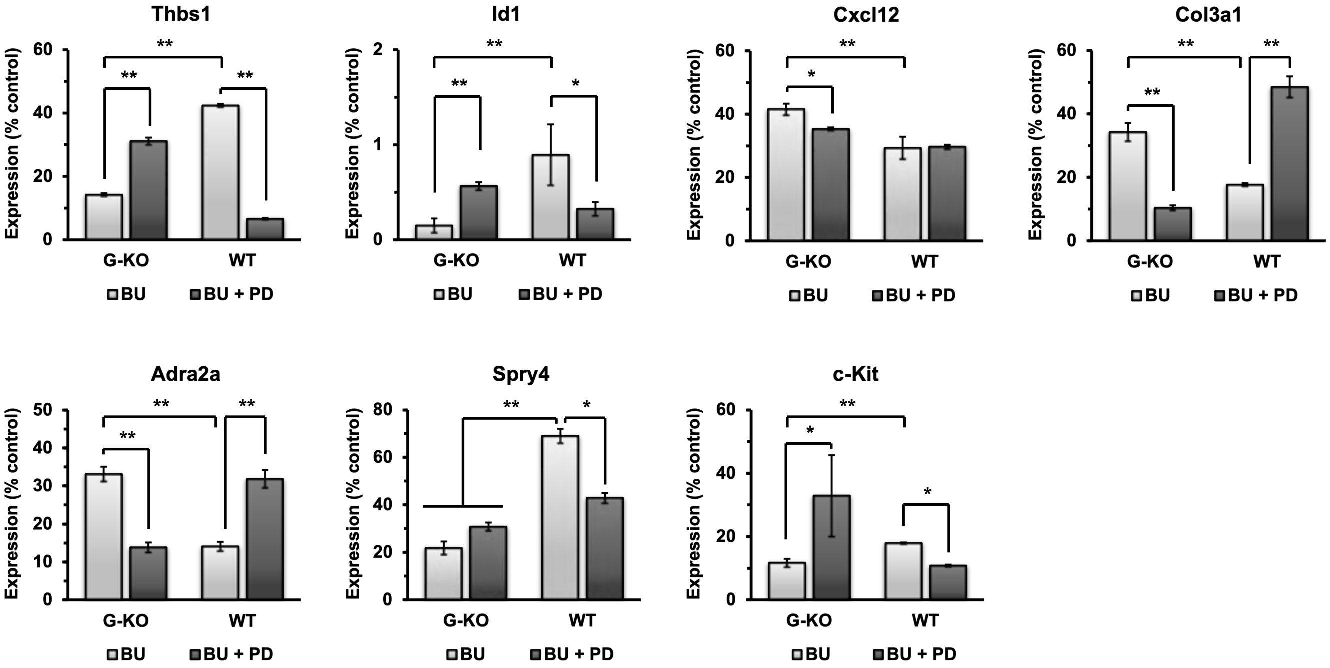

Supplementary Figure 5. qRT-PCR analysis of selected genes.

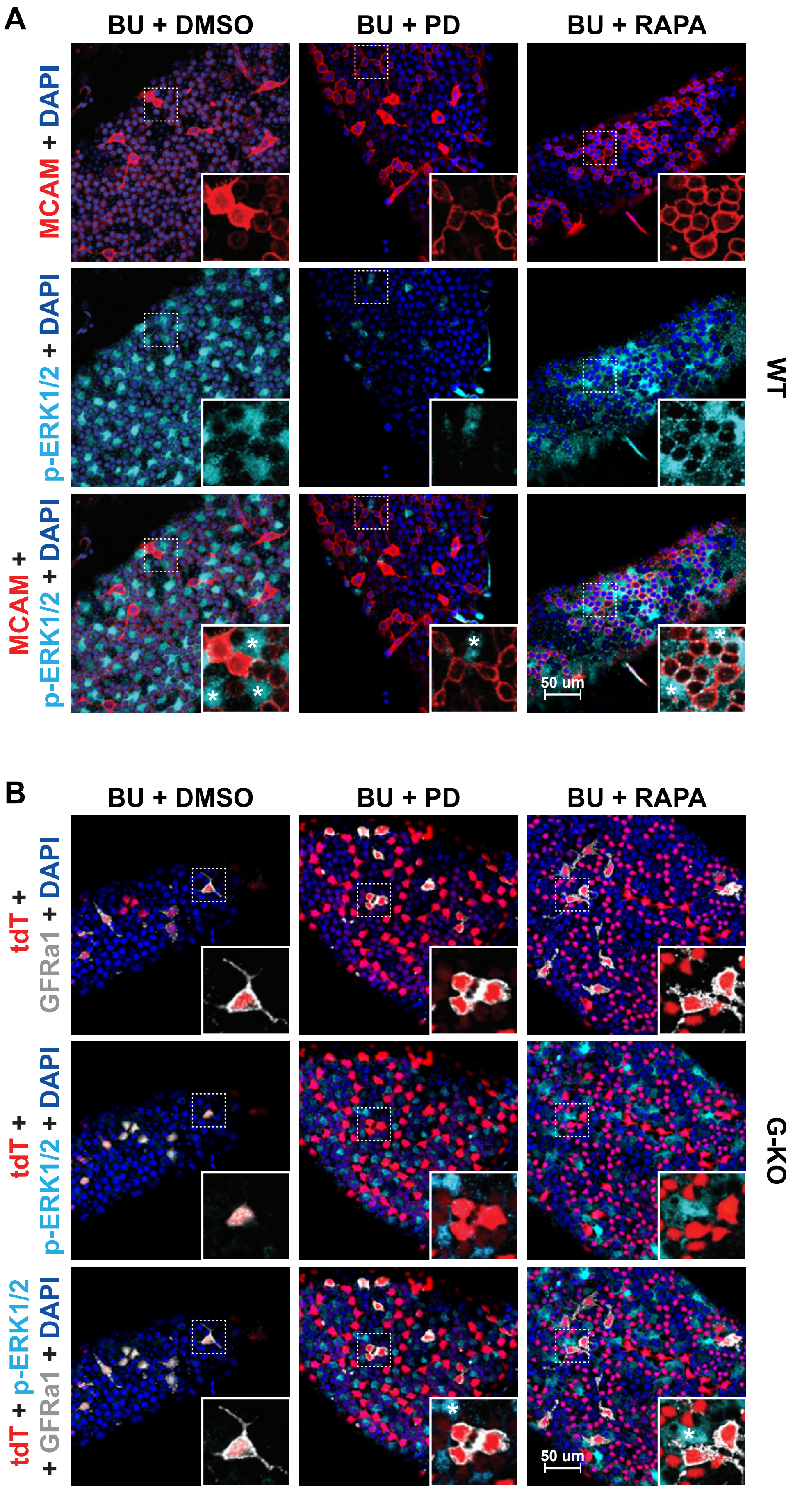

Supplementary Figure 6. ERK activity within the seminiferous tubules during regeneration (BU-D10).

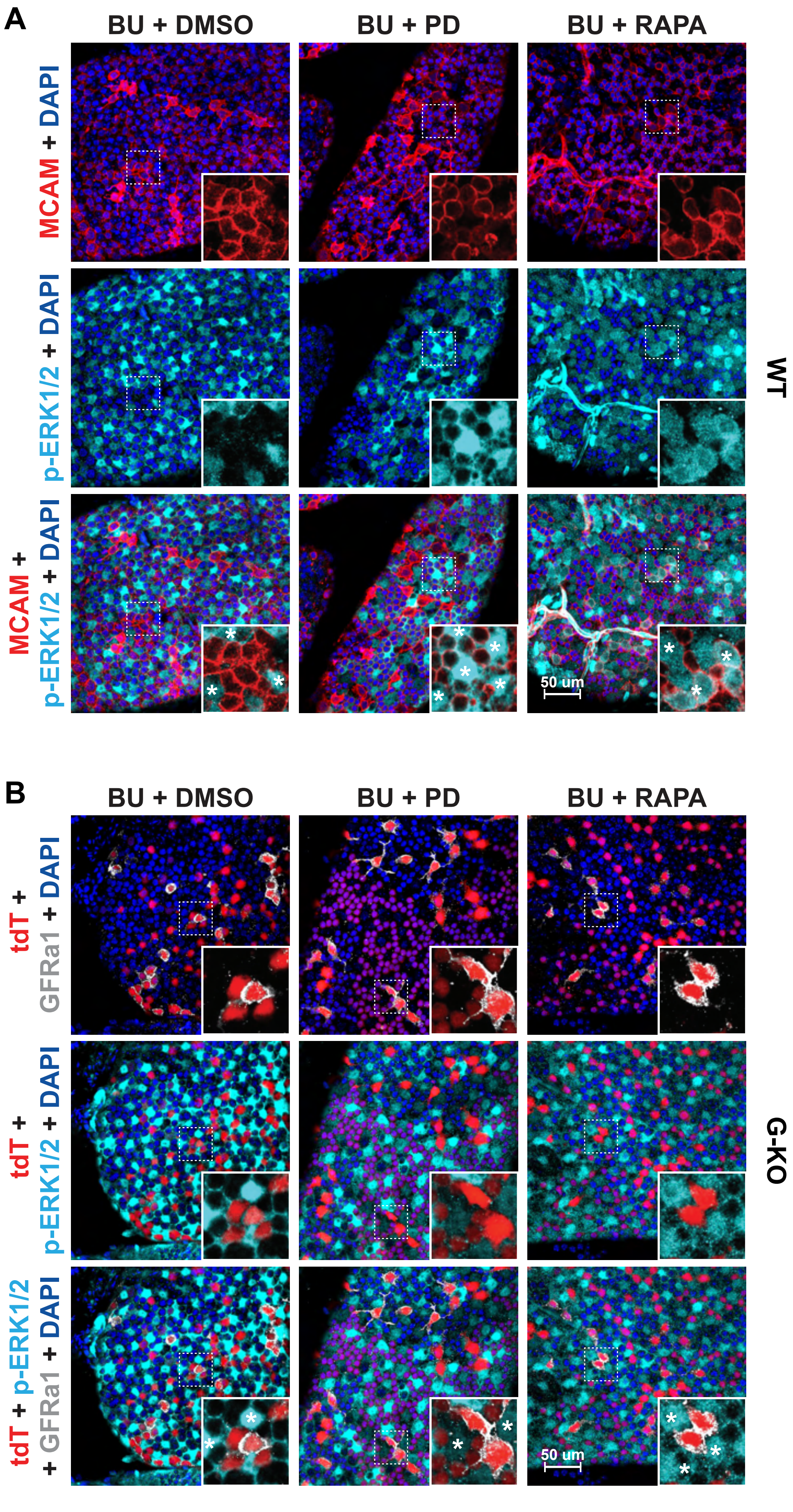

Supplementary Figure 7. ERK activity within the seminiferous tubules quickly after damage (BU-D2).

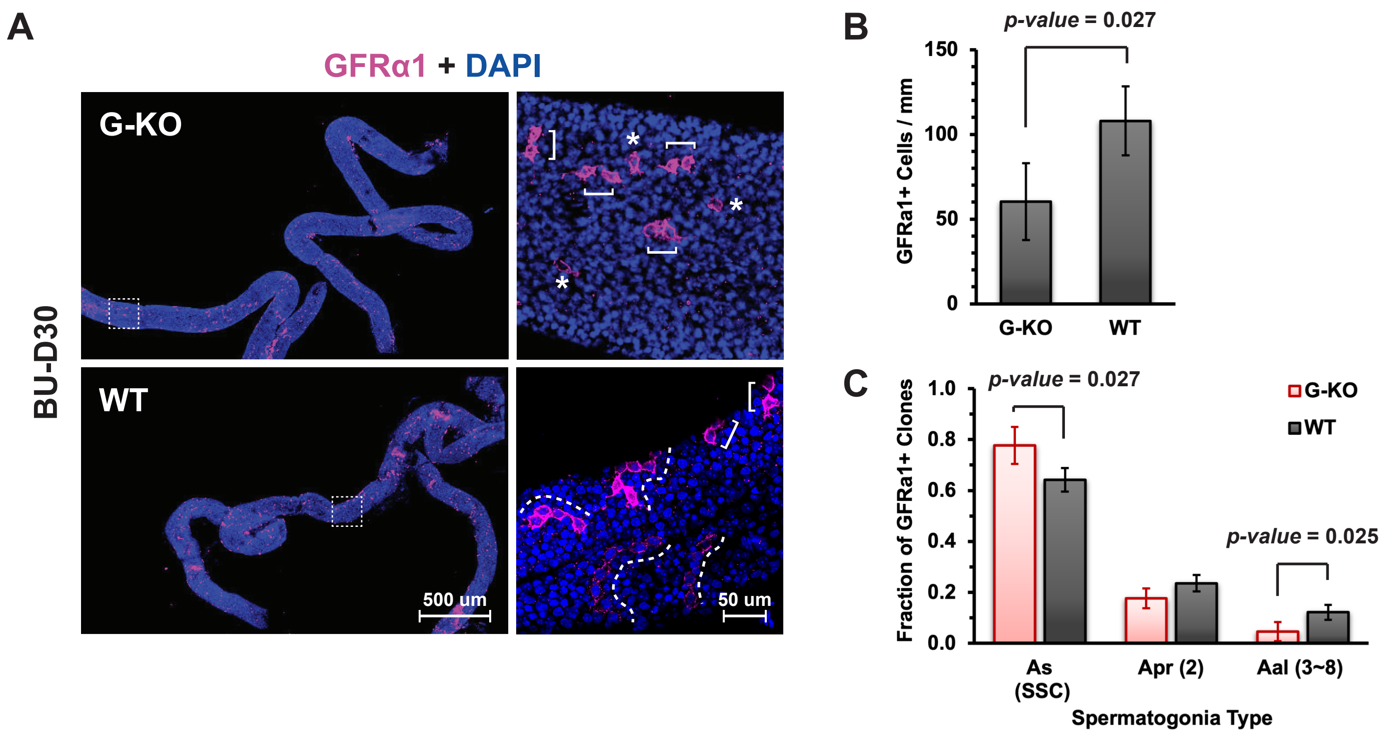

Supplementary Figure 8. Spermatogonia remain reduced in adult *Spry4*$^{G\text{-}KO}$ mice 30 days after damage (BU-D30).

Supplementary Table 1. Primer sequences for genotyping

Target	Primer	Sequence
Gfra1-creERT2	Fwd	CTTCCAGGTTGGGTCGGAACTGAACCC
	Rev	GTGAAACAGCATTGCTGTCACTT
Rosa26-LSL-tdTomato	Fwd-WT (oIMR9020)	AAGGGAGCTGCAGTGGAGTA
	Rev-WT (oIMR9021)	CCGAAAATCTGTGGGAAGTC
	Fwd-tdRFP (oIMR9105)	CTGTTCCTGTACGGCATGG
	Rev-WPRE (oIMR9103)	GGCATTAAAGCAGCGTATCC
Spry4	Fwd	CAGGACTTGGGAGTGCTTCCTTAG
	Rev-Floxed	CCTCCTAGTACCTTTTTGGGGAGAG
	Rev	TACAGCAGGAATGGCTACGGTG

Supplementary Table 2. Primer sequences for qRT-PCR

Target	Primer	Sequence
Adra2a	Fwd	CTTTTGCACGTCGTCCATAGT
	Rev	CGGTGACAATGATGGCCTTGA
Col3a1	Fwd	CTGTAACATGGAAACTGGGGAAA
	Rev	CCATAGCTGAACTGAAAACCACC
CXCL12	Fwd	GAGCCAACGTCAAGCATCTG
	Rev	CGGGTCAATGCACACTTGTC
Id1	Fwd	CCTAGCTGTTCGCTGAAGGC
	Rev	GTAGAGCAGGACGTTCACCT
Spry4	Fwd	TGCGTCAAGCTGGCCCAGCG
	Rev	CAGACCTGCTGGTCTTGGTG
Tbp	Fwd	GGGGAGCTGTGATGTGAAGT
	Rev	CCAGGAAATAATTCTGGCTCA
Thbs1	Fwd	GTGAGGTTTGTCTTTGGAACCA
	Rev	GTTGTTGTCAAGGGTAAGAAGGA
